# EpOMEs act as immune suppressors in a lepidopteran insect, *Spodoptera exigua*

**DOI:** 10.1038/s41598-020-77325-2

**Published:** 2020-11-19

**Authors:** Mohammad Vatanparast, Shabbir Ahmed, Dong-Hee Lee, Sung Hee Hwang, Bruce Hammock, Yonggyun Kim

**Affiliations:** 1 Department of Plant Medicals, Andong National University, Andong, 37629 South Korea; 2 Industry Academy Cooperation Foundation, Andong National University, Andong, 36729 South Korea; 3 Department of Entomology and Nematology, UC Davis Comprehensive Cancer Center, University of California, Davis, CA USA

**Keywords:** Immunology, Physiology

## Abstract

Epoxyoctadecamonoenoic acids (EpOMEs) are epoxide derivatives of linoleic acid (9,12-octadecadienoic acid) and include 9,10-EpOME and 12,13-EpOME. They are synthesized by cytochrome P450 monooxygenases (CYPs) and degraded by soluble epoxide hydrolase (sEH). Although EpOMEs are well known to play crucial roles in mediating various physiological processes in mammals, their role is not well understood in insects. This study chemically identified their presence in insect tissues: 941.8 pg/g of 9,10-EpOME and 2,198.3 pg/g of 12,13-EpOME in fat body of a lepidopteran insect, *Spodoptera exigua*. Injection of 9,10-EpOME or 12,13-EpOME into larvae suppressed the cellular immune responses induced by bacterial challenge. EpOME treatment also suppressed the expression of antimicrobial peptide (AMP) genes. Among 139 *S. exigua* CYPs, an ortholog (*SE51385*) to human EpOME synthase was predicted and its expression was highly inducible upon bacterial challenge. RNA interference (RNAi) of *SE51385* prevented down-regulation of immune responses at a late stage (> 24 h) following bacterial challenge. A soluble epoxide hydrolase (*Se-sEH*) of *S. exigua* was predicted and showed specific expression in all development stages and in different larval tissues. Furthermore, its expression levels were highly enhanced by bacterial challenge in different tissues. RNAi reduction of Se-sEH interfered with hemocyte-spreading behavior, nodule formation, and AMP expression. To support the immune association of EpOMEs, urea-based sEH inhibitors were screened to assess their inhibitory activities against cellular and humoral immune responses of *S. exigua*. 12-(3-adamantan-1-yl-ureido) dodecanoic acid (AUDA) was highly potent in suppressing the immune responses. The addition of AUDA to a pathogenic bacterium significantly increased bacterial pathogenicity by suppressing host immune defense. In sum, this study demonstrated that EpOMEs play a crucial role in facilitating anti-inflammatory responses in *S. exigua*.

## Introduction

Polyunsaturated fatty acids (PUFAs) are fatty acids that contain two or more double bonds in their backbone. The importance of PUFAs as essential fatty acids has been appreciated since the discovery that omega-3 and omega-6 fatty acids are required in the animal diet for viability^1^. In particular, long chain-PUFAs (LC-PUFAs: 20 or more carbon atoms) function as signaling molecules to mediate immunity, development, and reproduction^2^. LC-PUFAs are also rich (7–25%) in aquatic insects^3^, but are rare in terrestrial insects due to evolutionary adaptations to prevent oxidative stress^4^. However, terrestrial insects are rich in linoleic acid (LA: 18 carbon atoms) present in phospholipids (PLs), and this fatty acid might be available for the production of LC-PUFAs with the help of elongase and desaturase activities^5^.

Epoxyoctadecamonoenoic acids (EpOMEs) including vernolic acid (12,13-EpOME) and coronaric acid (9,10-EpOME) are produced by neutrophils and macrophages in mammals and are known to be leukotoxins^6,7^. EpOMEs are synthesized from LA by specific cytochrome P450 monooxygenases (CYPs) classified into CYP2J and CYP2C subfamilies^8^. Excessive production of EpOMEs has been associated with acute respiratory distress syndrome^9^. Subsequent study has shown that EpOME toxicity comes from their metabolites [9,10-dihydroxy-octadecamonoenoate (9,10-DiHOME) and 12,13-dihydroxy-octadecamonoenoate (12,13-DiHOME)] which are produced by the catalytic activity of soluble epoxide hydrolase (sEH)^10^.

EpOME and DiHOME were detected in all developmental stages of a mosquito, *Culex quinquefasciatus*^11^*.* Feeding sEH inhibitor increased EpOME levels in the mosquito midgut and reduced the total number of bacteria in the lumen, suggesting that EpOMEs are associated with insect immunity. However, it is not clear whether EpOMEs mediate typical cellular or humoral immune responses. Other epoxy fatty acids such as epoxyeicosatrienoic acids (EETs) derived from arachidonic acid were detected in a lepidopteran insect, *Spodoptera exigua*^12^*.* In that study, four different EETs mediated cellular and humoral immune responses by activating hemocyte behavior and inducing antimicrobial peptide (AMP) gene expression. Furthermore, Vatanparast et al.^12^ proposed that four CYPs are involved in EET biosynthesis. However, genetic factors related to EpOME biosynthesis have not been reported in insects although EpOMEs and EETs are synthesized by similar CYPs in mammals^13^.

This study aimed to address the immunological functions of EpOMEs in *S. exigua*. To achieve this, endogenous EpOME levels were measured in internal tissues of *S. exigua* using LC–MS/MS. To support the presence of EpOMEs, this study further predicted their biosynthetic and degradation enzymes. To test physiological functions associated with immunity, individual EpOMEs were injected into larvae and subsequent immunological changes were monitored. Finally, the immunological functions of EpOMEs were validated by treating insects with sEH inhibitor to elevate EpOME levels.

## Results

### Two EpOMEs identified in *S. exigua* fat body

**Figure 1:**
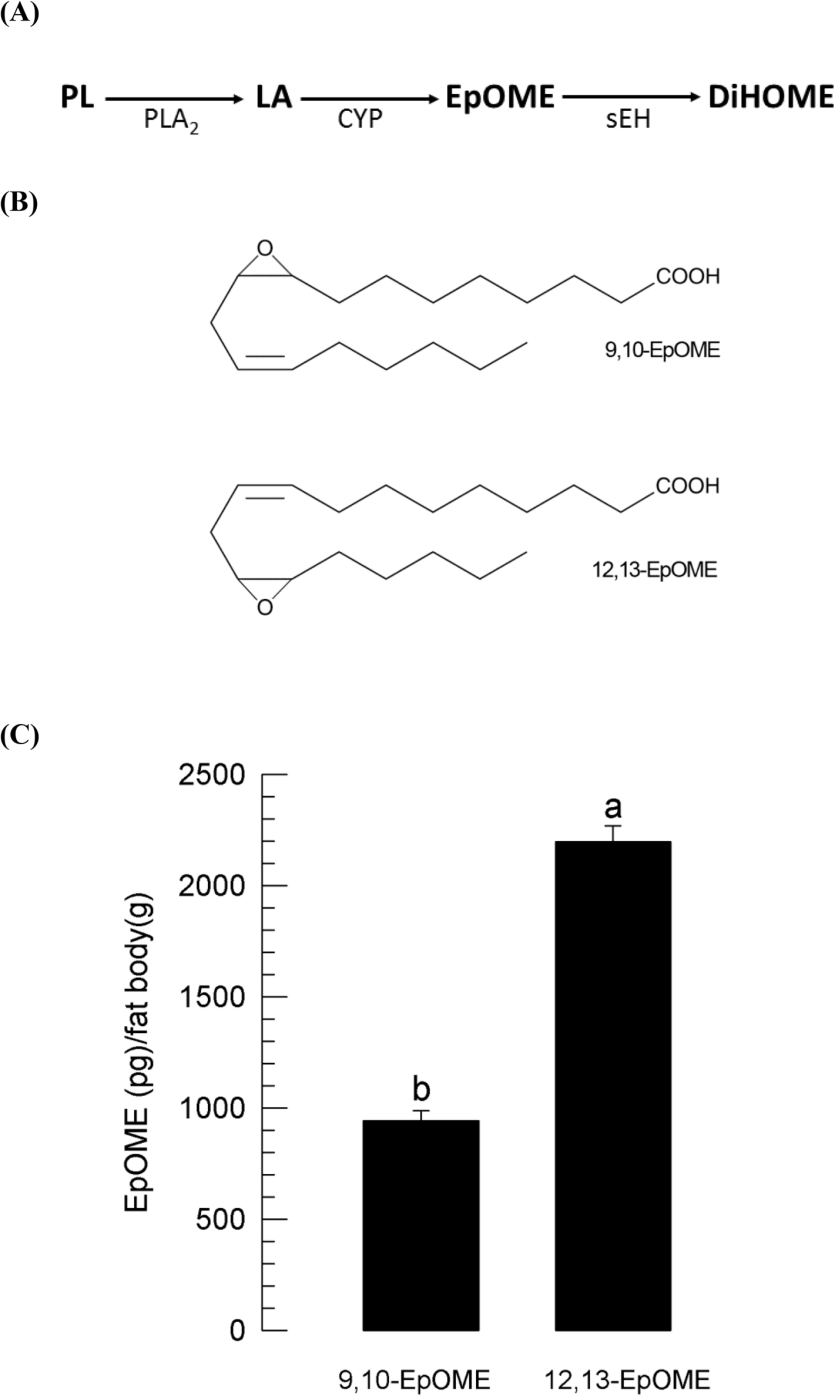
Detection of EpOMEs in larval fat body of *S. exigua*. (**A**) A hypothetical pathway of EpOME biosynthesis in *S. exigua*. Phospholipase A_2_ (PLA_2_) catalyzes phospholipid (PL) to release linoleic acid (LA), which is oxidized by cytochrome P450 monooxidase (CYP) to produce EpOME. The epoxy ring of EpOME is then hydroxylated by soluble epoxide hydrolase (sEH) to form dihydroxy-octadecamonoenoate (DiHOME). (**B**) Two EpOMEs: 9,10-EpOME and 12,13-EpOME (**C**) EpOME levels in fat body collected from larvae immunized with bacteria. L5 larvae were challenged with 5 × 10^5^ *E. coli* cells. After 8 h PI, fat body tissues were collected and used to measure EpOME levels using LC–MS/MS. Each EpOME assessment was replicated with three independent samples. Different letters above standard deviation bars indicate significant difference among means at Type I error = 0.05 (LSD test). Chromatograms of EpOME analyses are presented in Supplementary Fig. S1.

Due to LA-rich composition in *S. exigua* PLs^5^, a biosynthetic pathway for EpOMEs may be initiated from the catalytic activities of phospholipases including PLA_2_ (Fig. 1A). Free LA is then epoxydized largely by a specific CYP to generate two EpOMEs (Fig. 1B). These two EpOMEs can be degraded by sEH to the respective DiHOMEs. Two EpOMEs were detected in *S. exigua* using LC–MS/MS. To avoid any contamination from diet, fat body tissues were isolated to measure endogenous EpOMEs in L5 larvae of *S. exigua* (Supplementary Fig. S1). Two EpOMEs were detected in the larval fat bodies. Two different ion peaks were used for tandem mass analyses to confirm the identity of the EpOMEs based on retention times. EpOME standards confirmed the two found in the fat body samples at two ion peaks. *S. exigua* larvae had more 12,13-EpOME (2198.3 pg/g fat body) than 9,10-EpOME (941.8 pg/g fat body) (Fig. 1C).

### Suppressive effect of EpOMEs on hemocyte-spreading behavior

**Figure 2:**
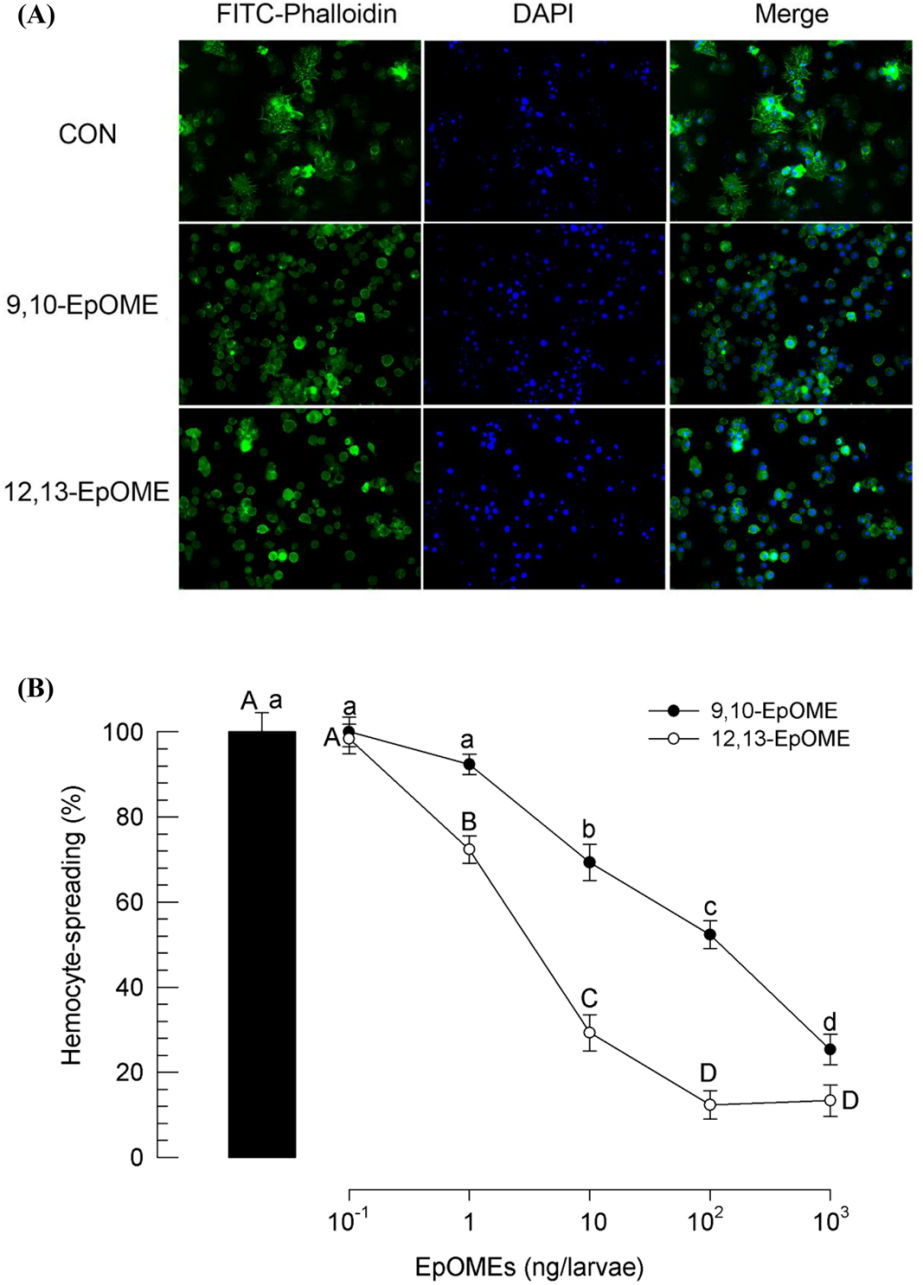
Suppressive effect of EpOMEs on hemocyte-spreading behavior in *S. exigua.* (**A**) Inhibition of F-actin growth in hemocytes of larvae injected with 3 µL of EpOME (1 µM). At 8 h PI, hemocytes were observed under a fluorescence microscope at ×400 magnification. F-actin was stained with FITC-phalloidin (green) while nucleus was stained with DAPI (blue). (**B**) Quantification of hemocyte-spreading behavior upon exposure to different doses of EpOMEs. Hemocyte-spreading behavior was quantitatively measured in 100 randomly chosen cells with three replications. Each treatment was replicated three times. Different letters above standard deviation bars indicate significant difference among means at Type I error = 0.05 (LSD test).

To test the hypothesis that EpOMEs are associated with insect immune responses, hemocyte-spreading behavior was assessed by F-actin growth detected by FITC-labeled phalloidin (Fig. 2A). In the control group, hemocytes showed active extension of the actin cytoskeleton. However, injection of 0.1 µg/larva of EpOMEs markedly prevented the F-actin growth. The suppressive effects of the two EpOMEs on hemocyte-spreading behavior followed a dose-dependent relationship (Fig. 2B). 12,13-EpOME was the more potent inhibitor of hemocyte behavior (*F* = 37.56; *df* = 1, 20; *P* < 0.0001).

### Suppressive activities of EpOMEs against cellular and humoral immune responses

**Figure 3:**
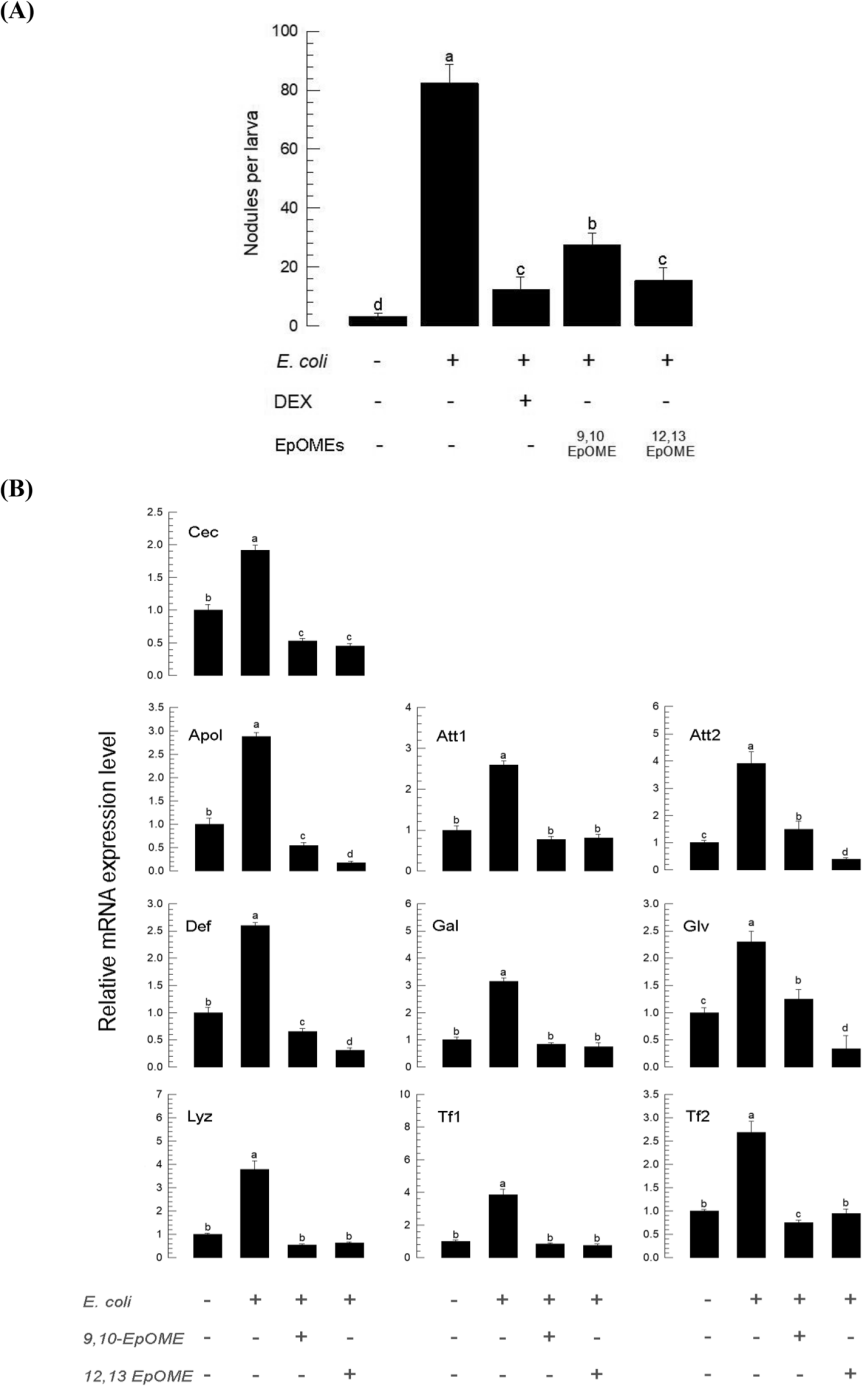
Suppressive effect of EpOMEs on immune responses of *S. exigua*. (**A**) Inhibitory activity of EpOME treatment (0.1 µg/larva) on cellular immune response measured by nodule formation following bacterial challenge (*E. coli* injection, 5 × 10^5^ cells per larva). A PLA_2_ inhibitor, dexamethasone (ʻDEXʼ, 1 µg/larva), was used as a negative control. (**B**) Inhibitory activity of EpOME treatment (0.1 µg/larva) against humoral immune response measured by expression levels of 10 AMP genes including apolipophorin III (ʻApolʼ), attacin 1 (ʻAtt1ʼ), attacin 2 (ʻAtt2ʼ), cecropin (ʻCecʼ), defensin (ʻDefʼ), gallerimycin (ʻGalʼ), gloverin (ʻGlvʼ), lysozyme (ʻLyzʼ), transferrin 1 (ʻTf1ʼ), and transferrin 2 (ʻTf2ʼ). Each treatment was replicated three times. Different letters indicate significant differences among means at Type I error = 0.05 (LSD test).

The inhibitory activity of EpOMEs on hemocyte behavior suggested that they were capable of exerting negative control on cellular immune responses. To test this hypothesis, hemocyte nodule formation in response to bacterial challenge was assessed by the injection of EpOMEs in larvae of *S. exigua* (Fig. 3A). *S. exigua* larvae formed over 80 nodules per larva eight h after bacterial injection. However, the treatment with one μg of a PLA_2_ inhibitor (dexamethasone) per larva significantly (*P* < 0.05) reduced nodule formation. When EpOMEs were injected into larvae (0.1 μg per larva), they exhibited significant (*P* < 0.05) inhibitory effects on the cellular immune response, with 12,13-EpOME exhibiting greater inhibitory activity than 9,10-EpOME.

The inhibitory activities of EpOMEs were assessed in the humoral immune response of *S. exigua* by measuring AMP expression levels following bacterial challenge (Fig. 3B). Expression of 10 AMP genes were highly inducible to the immune challenge. However, EpOME significantly (*P* < 0.05) suppressed this induction for all ten AMPs. In particular, 12,13-EpOME suppressed expression of *cecropin*, *apolipophorin III*, *attacin 2*, *defensin*, and *gloverin* to much lower levels than those by 9,10-EpOME (*P* < 0.05).

### Prediction of EpOME synthase in *S. exigua*

**Figure 4:**
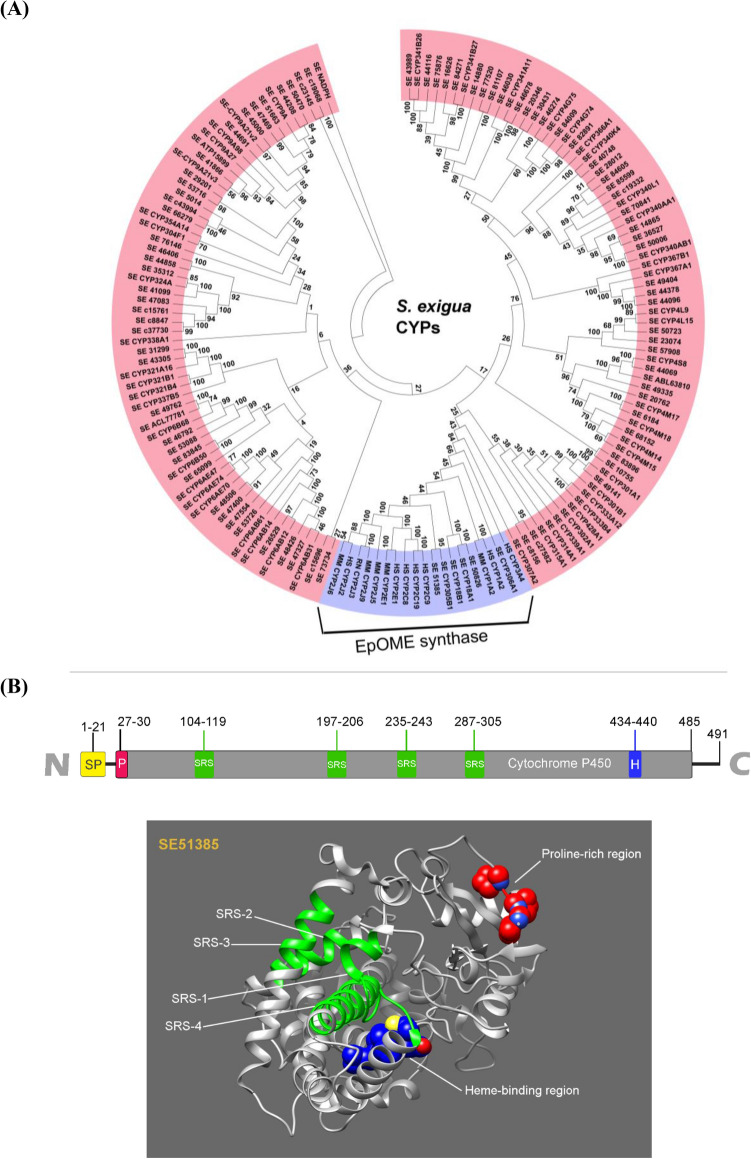
Prediction of EpOME synthase in *S. exigua*. (**A**) A phylogenetic tree of 139 cytochrome P450 oxidases (CYPs) of *S. exigua* and two mammalian EpOME synthases, CYP2J6 of *M. musculus* and CYP2J2 of *H. sapiens*. Tree was generated with MEGA 7 using Neighbor-joining method. Bootstrap values on nodes were obtained with 1000 replicates. GenBank accession numbers of all sequences are listed in Supplementary Table S1. (**B**) Functional domains of SE51385, in which yellow, pink, green and blue regions indicate signal peptide (‘SP’), proline-rich region (‘P’), four substrate recognition sites (‘SRS1-4’), and heme-binding (‘H’), respectively. The domains of SE51385 are demonstrated with three dimensional analysis.

Using CYP2C8, CYP2J2 and CYP2J6 sequences of mammals that have epoxygenase activity(Moran et al., 2000; Hanif et al., 2016), 139 annotated CYPs of *S. exigua* were interrogated to predict orthologs encoded in the *S. exigua* transcriptome (Fig. 4A). The mammalian epoxygenases shared sequence homology with eight *S. exigua* CYPs in a monophyletic cluster. This gene cluster includes three SeCYPs (*CYP18A1*, *CYP18B1*, and *CYP305B1*) associated with EET synthesis^12^. Interestingly, *SE51385* and SE50826 are closely related to the mammalian EpOME synthases. In particular, they share high identities (E values = 3e−79 and 2e−87, respectively) with CYP2C8 of *Homo sapiens*. However, SE50826 is highly homologous (84.84%) to farnesoate epoxygenase of *Bombyx mori* while SE51385 shows the sequence homology only at 69.12%. Thus, SE51385 was considered to be a candidate of EpOME synthase among the clustering members. Domain analysis of SE51385 (GenBank accession number: MT375603) encoding 491 amino acids showed that it shares heme-binding regions, three different proline-rich functional domains and four substrate recognition sites (SRSs) with mammalian EpOME synthases (Supplementary Fig. S2). The four SRSs are predicted to form α-helices around the heme-binding region (Fig. 4B). Containing a signal peptide, SE51385 was predicted to be glycosyl phosphatidylinositol (GPI) anchored to the membrane.

### Functional association of SE51385 with *S. exigua* immunity

**Figure 5:**
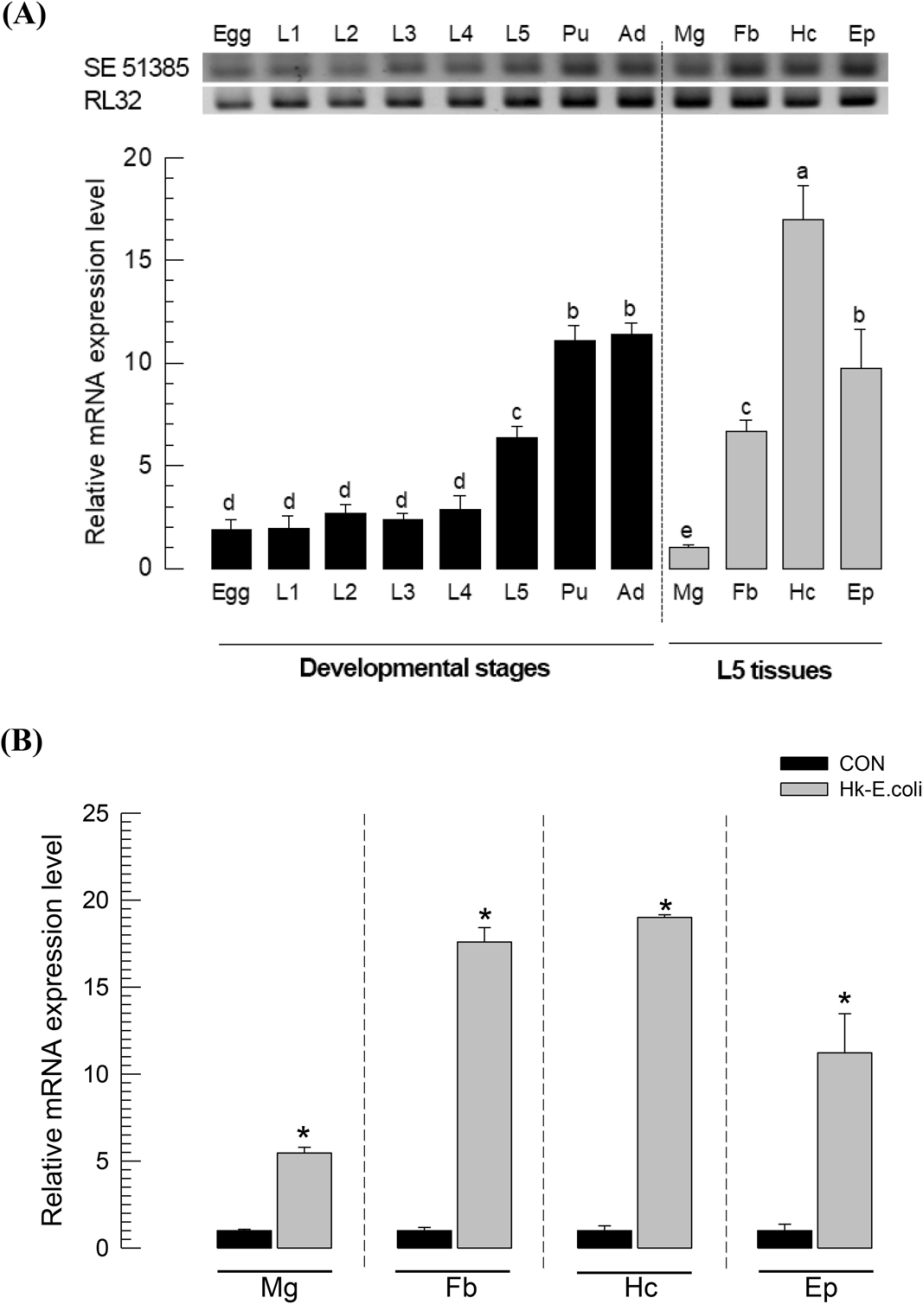
Expression profile of *SE51385* in *S. exigua*. (**A**) Expression in development [egg (‘Egg’), five larval instars (‘L1–L5’), pupa (‘Pu’), adult (‘Ad’)] and larval tissues [midgut (‘Mg’), fat body (‘Fb’), hemocytes (‘Hc’), and epidermis (‘Ep’)]. (**B**) Inducible expression levels of *SE51385* in response to bacterial challenge with heat-killed *E. coli* (‘Hk-E.coli’, 5 × 10^5^ cells per L5 larva) in different tissues*. RL32* was used to normalize the expression levels in different RT-qPCR samples. Each treatment was replicated three times. Different letters or stars above standard deviation bars indicate significant difference among means at Type I error = 0.05 (LSD test).

*SE51385* was expressed in all developmental stages from egg to adult (Fig. 5A). In L5 larvae, it was highly expressed in hemocytes as well as in other tissues such as epidermis, fat body, and midgut. *SE51385* expression was inducible to bacterial challenge in all four tissues (Fig. 5B).

**Figure 6:**
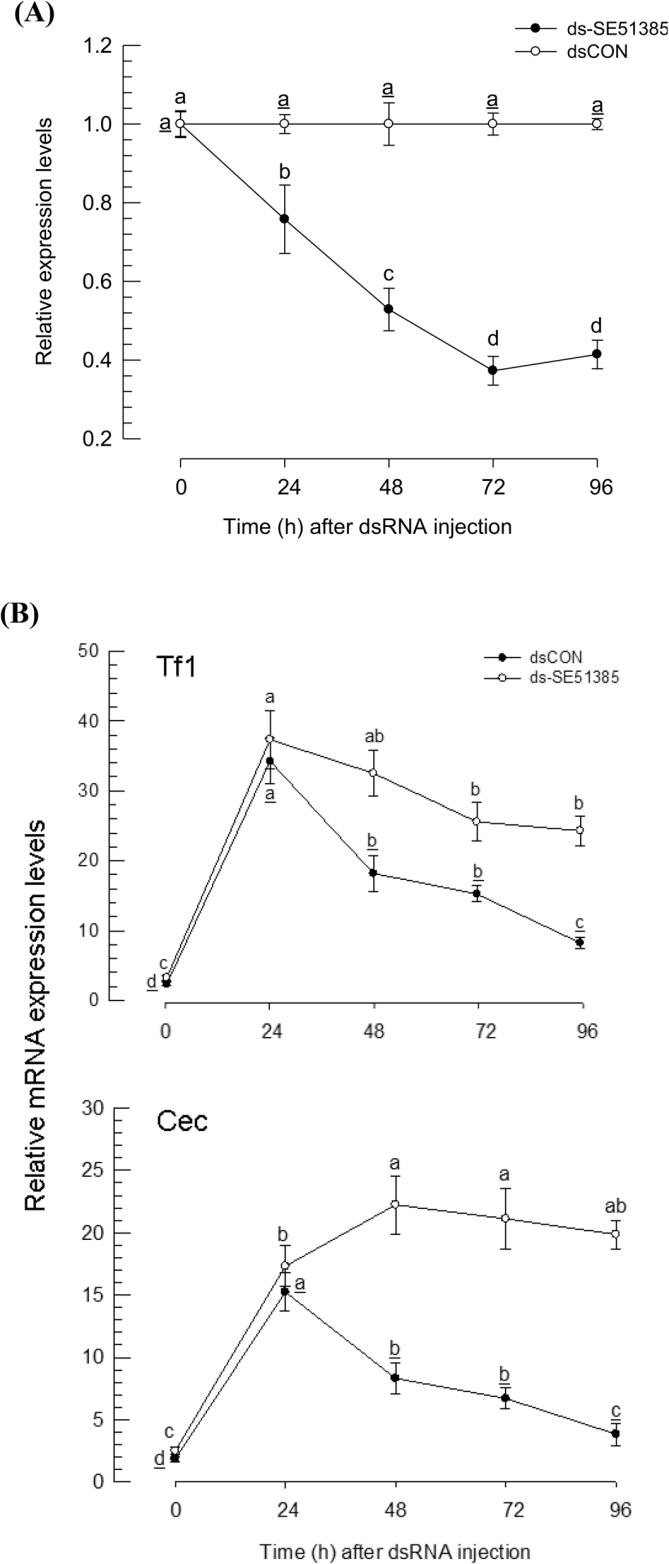
Suppressive effect of SE51385 on expression of AMP genes in *S. exigua*. (**A**) RNA interference (RNAi) of *SE51385* expression by hemocoelic injection of double-stranded RNA (ds-SE51385) to *S. exigua* larvae. dsRNA specific to *SE51385* (~ 400 bp) was constructed and injected to each L4 larva at 1 μg. *SE51385* mRNA levels were relatively quantified by RT-qPCR. Each treatment was replicated with three different insects. (**B**) Effect of RNAi on expression levels of two AMP genes: *transferrin 1* (‘Tf1’) and *cecropin* (‘Cec’). The mRNA levels in each test sample were normalized by respective *RL32* expression level. Control RNAi (‘dsCON’) was injected with dsRNA specific to *GFP* gene. Each treatment was replicated three times. Different letters above standard deviation bars indicate significant difference among means at Type I error = 0.05 (LSD test).

RNAi downregulation of *SE51385* expression was performed by injecting gene-specific dsRNA to L4 larvae (Fig. 6A). After injection of 1 μg of dsRNA to larva, a significant reduction in *SE51385* was seen from 24 to 96 h post-injection (PI) (*P* < 0.05). To test whether a functional association of the immune-mediated inducible expression exists, gene expression of two AMPs were monitored (Fig. 6B). Both AMP genes were highly inducible to the bacterial challenge. However, their expression levels significantly declined by 24 h PI (*P* < 0.05). Interestingly, *SE51385*-specific RNAi treatment prevented the reduction in AMP expression after 24 h induction. Especially, *cecropin* expression showed no significant decrease even at 96 h (*P* < 0.05).

### Prediction of sEH gene from *S. exigua* transcriptome

**Figure 7:**
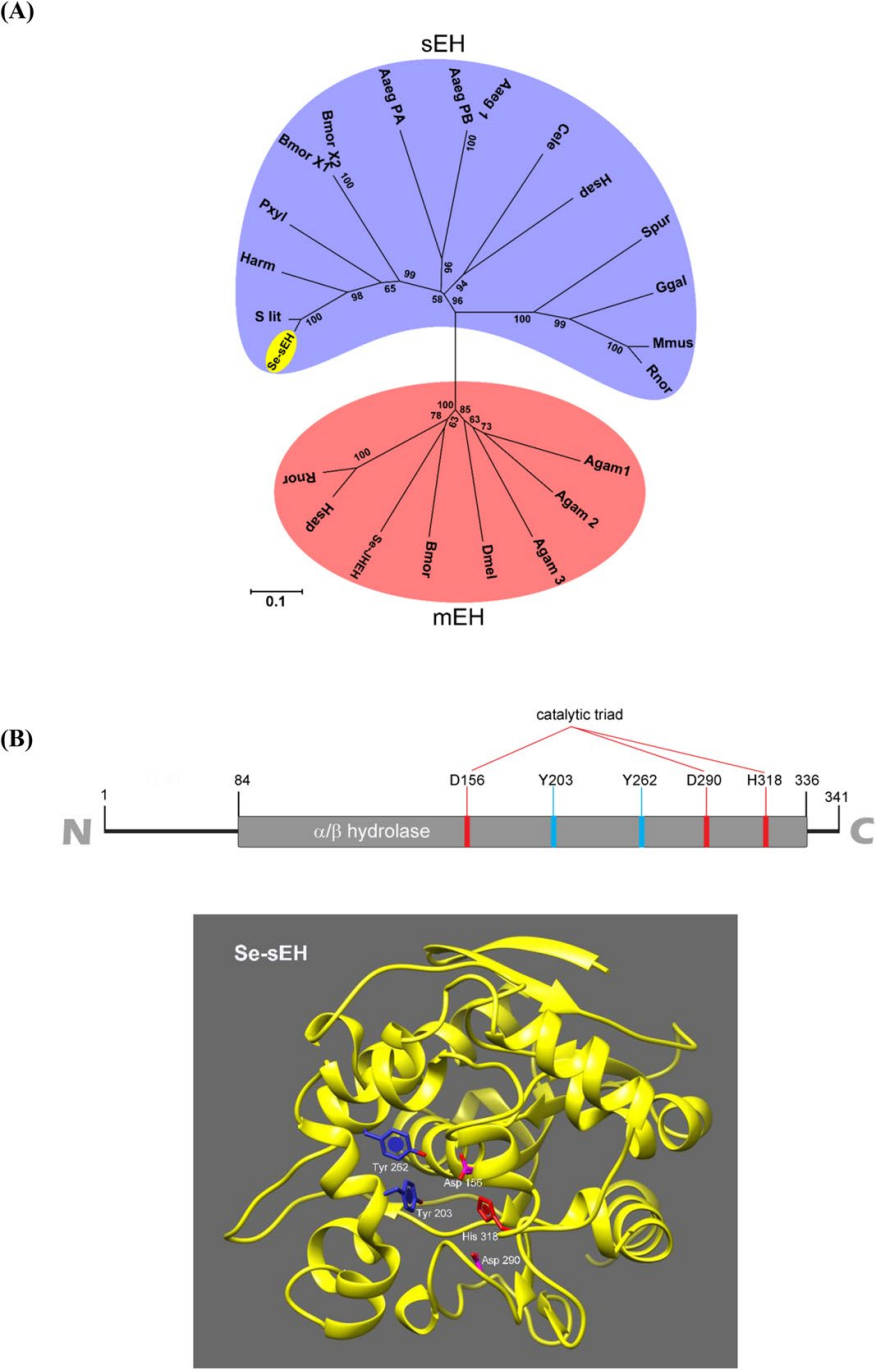

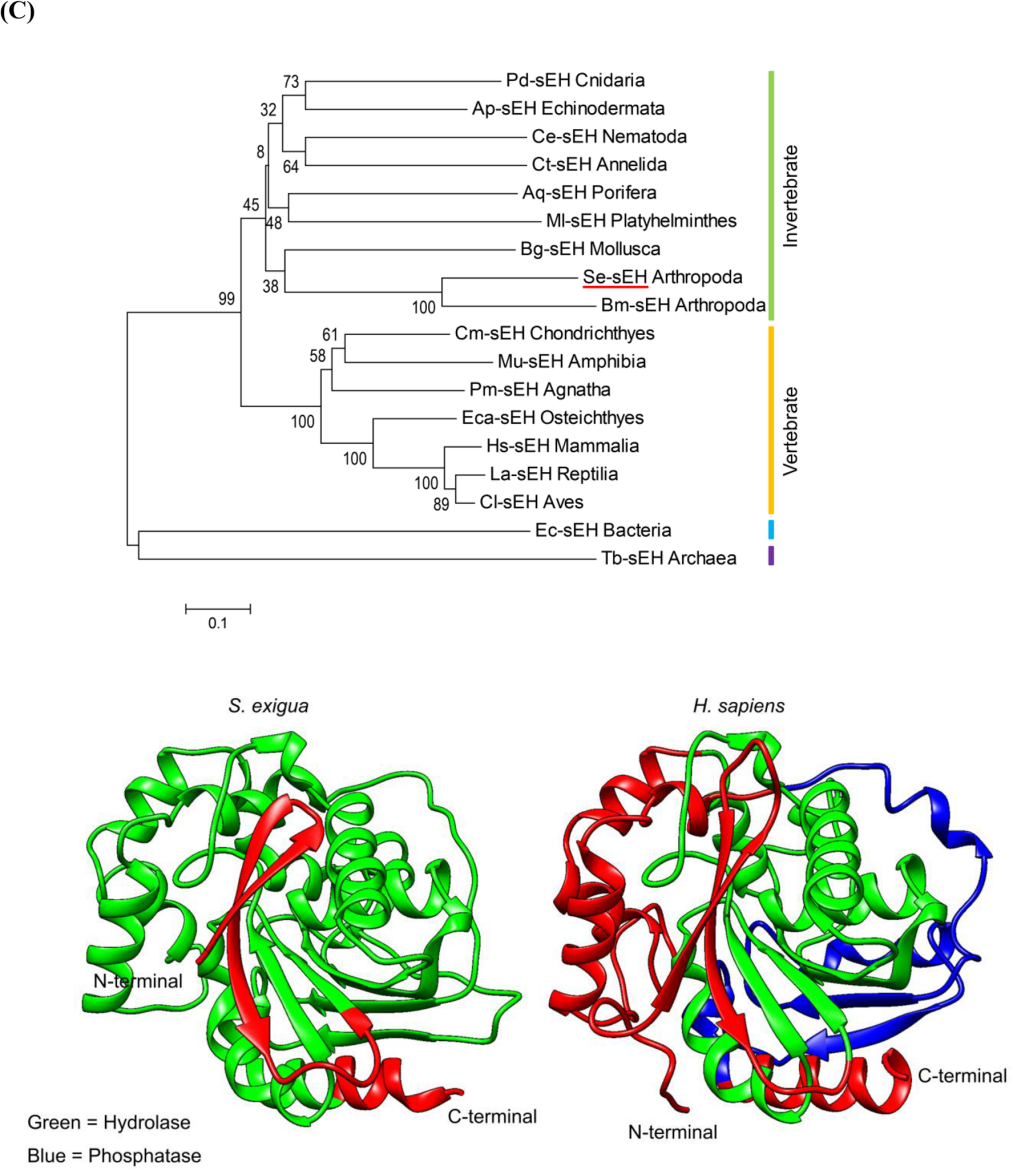
Molecular characterization of *S. exigua* soluble epoxide hydrolase (Se-sEH). (**A**) A phylogenetic tree of Se-sEH (yellow oval) with other sEHs and microsomal EHs (mEHs). The tree was generated with MEGA 7 using the neighbor-joining method. Nodes with > 50% bootstrap values (1000 replicates) are indicated on branches. (**B**) Prediction of functional domains of Se-sEH. Catalytic triad (Asp156, His318, and Asp290) around Tyr203 and Tyr262. (**C**) No additional N-terminal phosphatase domain in Se-sEH by phylogenetic and three dimensional comparisons with other sEHs. GenBank accession numbers of all sequences are listed in Supplementary Table S2. Comparison of domains between Se-sEH and *Homo sapiens* sEH (Hs-sEH). The domains were predicted using Pfam (https://pfam.xfam.org) and Prosite (https://prosite.expasy.org/). Three dimensional structures were prepared using UCSF Chimera (https://www.cgl.ucsf.edu/chimera/) where green and blue regions indicated hydrolase and phosphatase domains, respectively.

To predict a degradation pathway of EpOMEs to DiHOMEs, sEH ortholog(s) were screened from the hemocyte transcriptome (SRX259774) of *S. exigua* using the cDNA sequence (GenBank: ETN62858.1) of *Anopheles darlingi* sEH. Four partial contigs were matched (E value < 10^–10^) and assembled to be a full sequence (1418 bp, GenBank accession number: MT375604) containing an ORF encoding 341 amino acids. The ORF sequence (*Se-sEH*) was clustered with other sEHs and separated from microsomal EHs including juvenile hormone epoxide hydrolase of *S. exigua* (*Se-JHEH*) (Fig. 7A). The predicted amino acid sequence matched to the α/β hydrolase gene family containing the catalytic triad of Asp156, Asp290 and His318 around Tyr203 and Tyr262 (Fig. 7B, Fig. S3). sEHs are conserved in prokaryotes and eukaryotes (Fig. 7C) and invertebrate sEHs are distinct from vertebrate orthologs. Unlike mammalian sEHs, Se-sEH does not have an N-terminal phosphatase domain.

### Expression profile of Se-sEH in *S. exigua*

**Figure 8:**
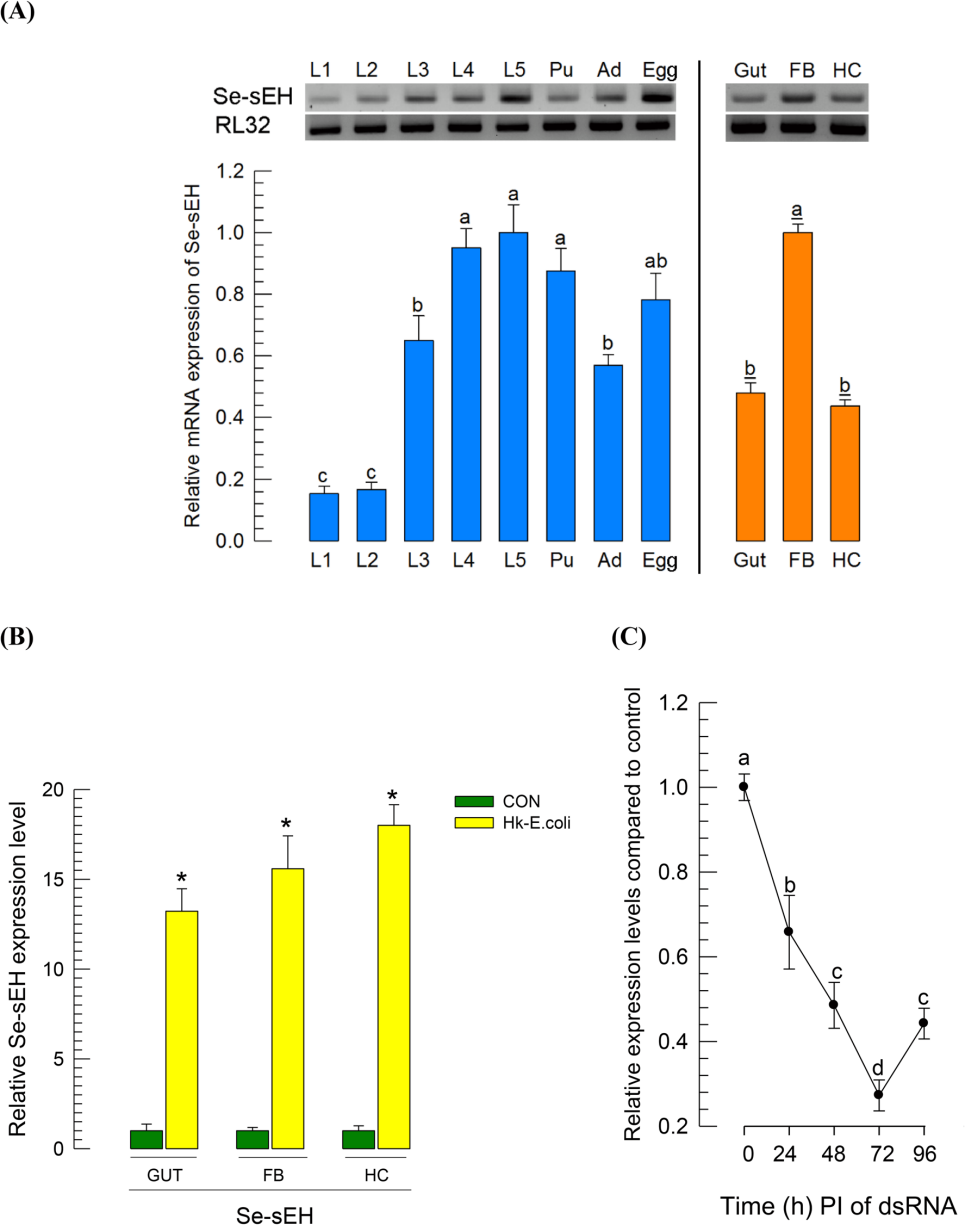
Expression profile of *S. exigua* soluble epoxide hydrolase (*Se-sEH*). (**A**) Expression patterns in different developmental stages and different larval tissues. Different developmental stages include five larval instars (‘L1–L5’), pupa (‘Pu’), adult (‘Ad’), and egg (‘Egg’). Different tissues of L5 larvae include midgut (‘Gut’), fat body (‘FB’), and hemocytes (‘HC’). (**B**) Inducible expression of *Se-sEH* in response to bacterial challenge with heat-killed *E. coli* (‘Hk-E.coli’, 5 × 10^5^ cells per L5 larva)*.* (**C**) RNA interference (RNAi) of *Se-sEH* using hemocoelic injection of double-stranded RNA (dsRNA) to *S. exigua* larvae. dsRNA specific to *Se-sEH* (354 bp) was constructed and injected into each L4 larva at 1 μg. At 24, 48, 72 and 96 h post injection, *Se-sEH* mRNA level was measured by RT-qPCR. mRNA levels were normalized by *RL32* expression level. Control RNAi (‘dsCON’) was injected with dsRNA specific to *GFP* gene. Each treatment was replicated with three different insects. Different letters and asterisks above standard deviation bars indicate significant difference among means at Type I error = 0.05 (LSD test).

The predicted *Se-sEH* was expressed in all *S. exigua* developmental stages tested, from egg to adult (Fig. 8A). However, its expression level varied among developmental stages, with high expression levels at L4, L5 and pupal stage. In larval stage, it was expressed in midgut, fat body and hemocytes. Its expression in larvae was inducible in response to immune challenge in all test tissues (Fig. 8B). RNAi of *Se-sEH* expression was performed by injecting gene-specific dsRNA to L4 larvae (Fig. 8C). When one μg of dsRNA was injected into each larva, *Se-sEH* expression was reduced (*P* < 0.05). The degree of suppression increased with increasing incubation time after dsRNA injection. The highest RNAi effect was observed at 72 h PI, exhibiting ~ 75% reduction in mRNA expression levels.

### Functional association of Se-sEH with immunity

**Figure 9:**
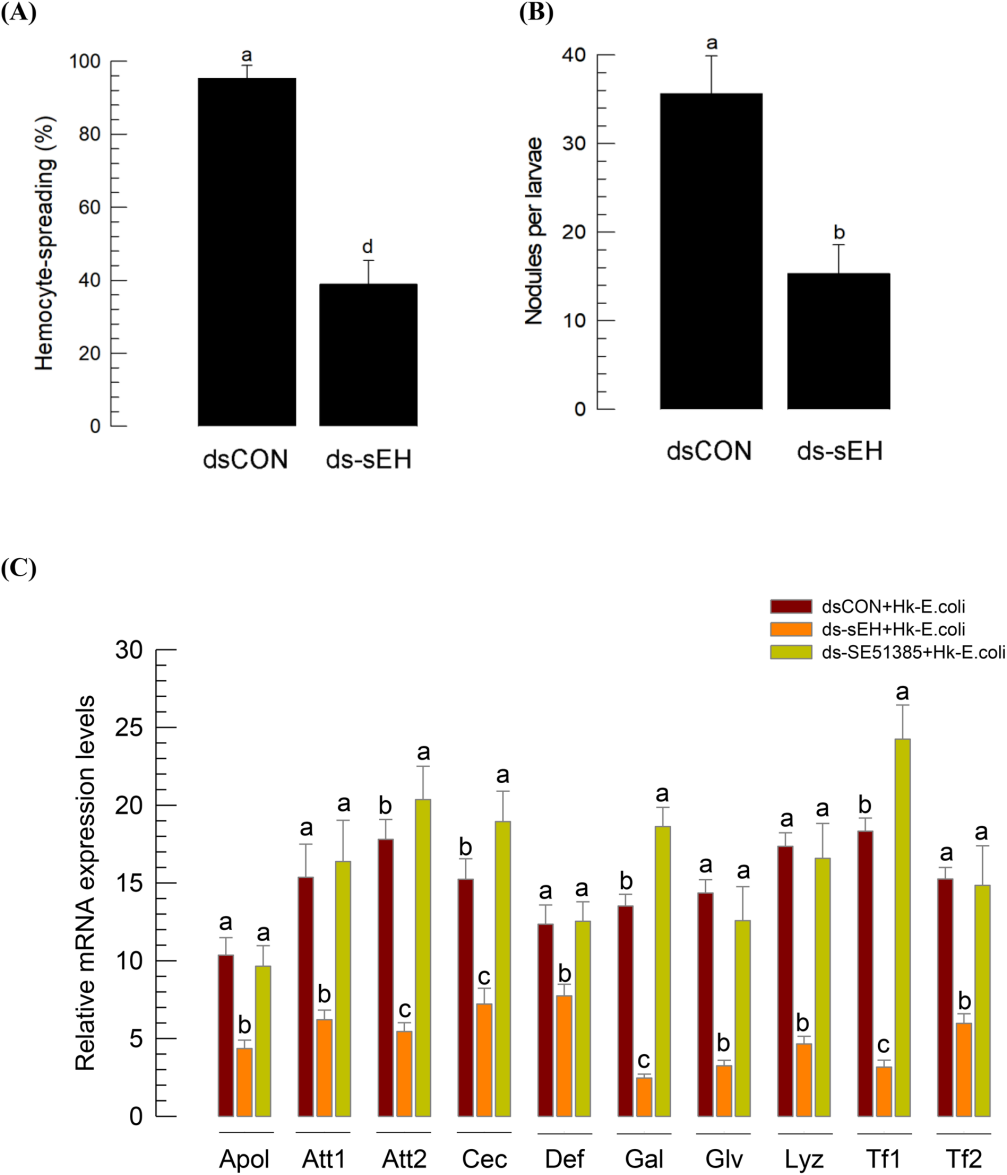
Effect of *Se-sEH* expression on immune responses of *S. exigua*. (**A**) Suppressive effects of *Se-sEH* RNAi on hemocyte-spreading behavior. RNAi was performed by injecting gene-specific dsRNA (ds-sEH) at 1 μg per larva. At 48 h PI, hemocytes were observed under a fluorescence microscope at ×400 magnification to count spread cells exhibiting outgrowth of F-actin filaments stained with FITC-phalloidin. Hemocyte-spreading behavior was quantitatively assessed in 100 randomly chosen cells with three replications. Control RNAi (‘dsCON’) was injected with dsRNA specific to *GFP* gene. (**B**) Suppressive effect of *Se-sEH* RNAi on nodule formation after injection of *E. coli* (5 × 10^5^ cells per larva). (**C**) Effects of RNAi to *Se-sEH* (ds-sEH) and SE51385 (ds-SE51385) on expression of ten AMP genes: *apolipophorin III* (ʻApolʼ), *attacin 1* (ʻAtt1ʼ), *attacin 2* (ʻAtt2ʼ), *cecropin* (ʻCecʼ), *defensin* (ʻDefʼ), *gallerimycin* (ʻGalʼ), *gloverin* (ʻGlvʼ), *lysozyme* (ʻLyzʼ), *transferrin 1* (ʻTf1ʼ), and *transferrin 2* (ʻTf2ʼ). For bacterial challenge, heat-killed *E. coli* (5 × 10^5^ cells per larva) were injected into larvae at 48 h after dsRNA treatment. At 8 h PI of bacteria, mRNA expression levels in fat body were assessed by RT-qPCR. *RL32* was used as an internal control. Each treatment was independently replicated three times. Different letters indicate significant differences among means at Type I error = 0.05 (LSD test).

RNAi downregulation of *Se-sEH* expression significantly suppressed hemocyte-spreading behavior (*P* < 0.05, Fig. 9A). This adverse effect on hemocyte behavior led to significant inhibition of cellular immune responses as measured by nodule formation following bacterial challenge (*P* < 0.05, Fig. 9B). Humoral immune responses influenced by *Se-sEH* were assessed by quantifying expression levels of ten AMP genes (Fig. 9C). All ten were highly inducible to bacterial injection. However, RNAi prevented up-regulation (*P* < 0.05). In contrast, RNAi treatment of *SE51385* expression did not suppress AMP gene expression. Interestingly, the RNAi treatment further upregulated expression of *attacin 2*, *cecropin*, *gallerimycin*, and *transferrin 1*.

### Effect of sEH inhibitors on *S. exigua* immune responses

**Figure 10:**
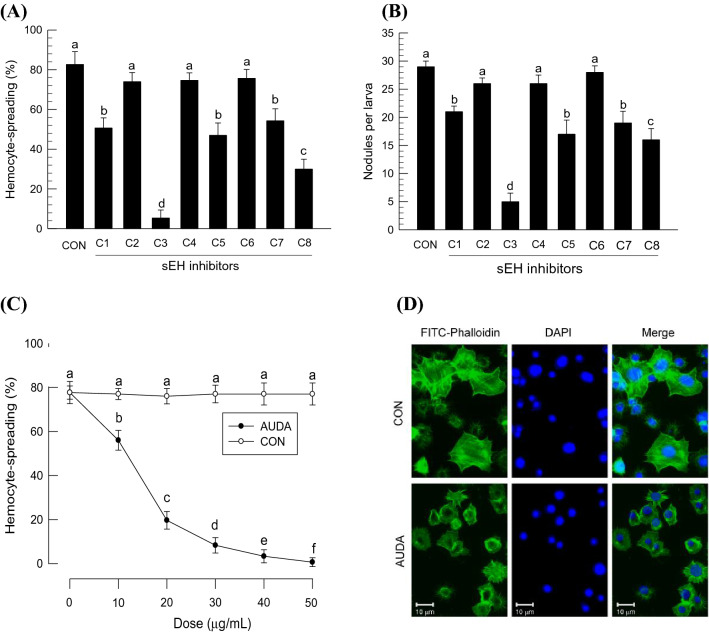
Inhibitory effects of sEH inhibitors on *S. exigua* immunity. The inhibitors include TPPU (ʽC1ʼ), PTUPB (ʽC2ʼ), AUDA (ʽC3ʼ), AEPU (ʽC4ʼ), *t*-AUCB (ʽC5ʼ), *t*-TUCB (ʽC6ʼ), *c*-TUCB (ʽC7ʼ), and AUCB (ʽC8ʼ). Control treatment (‘CON’) used vehicle solvent (DMSO). (**A**) Inhibitor effect on hemocyte-spreading behavior. Each inhibitor was injected into larvae at a dose of 0.1 µg/larva. At 8 h PI, hemocytes were counted under a fluorescence microscope at ×400 magnification. (**B**) Inhibitor effect on nodule formation in response to *E. coli* (5 × 10^5^ cells per larva). Each inhibitor was injected (0.1 µg/larva) along with bacteria. At 8 h PI, nodules were counted. (**C**) Dose–response of C3 (‘AUDA’) on inhibition of hemocyte-spreading behavior using in vitro assay. Each reaction mixture consisted of 50 μL of hemocyte suspension. (**D**) Inhibition of F-actin growth by AUDA treatment (0.1 µg/larva). At 8 h PI, hemolymph was collected and hemocytes were observed under a fluorescence microscope at ×400 magnification. F‐actin was observed by FITC‐phalloidin (green) while nuclei were stained with DAPI (blue). Each treatment was independently replicated three times. Different letters indicate significant differences among means at Type I error = 0.05 (LSD test).

Urea-based derivatives (Supplementary Fig. S4) as sEH inhibitors were used to confirm the physiological roles of *Se-sEH* in mediating cellular immune responses of *S. exigua* (Fig. 10). Among them, five compounds were able to effectively suppress hemocyte-spreading behavior (Fig. 10A). The suppressive effects were confirmed by assessing hemocyte-nodule formation following bacterial challenge (Fig. 10B). AUDA (‘C3’ in the figure) was the most effective. AUDA was further analyzed in dose response experiments (Fig. 10C) and could be seen to inhibit the hemocyte behavior in a range of 0 ~ 50 µg/mL. When only 0.1 µg of AUDA was injected, the larvae lacked hemocyte-spreading behavior upon immune challenge (Fig. 10D). AUDA also suppressed AMP gene expression of *S. exigua* in response to bacterial challenge (Supplementary Fig. S5).

### Potentiation of an entomopathogen by the addition of sEH inhibitors

**Figure 11:**
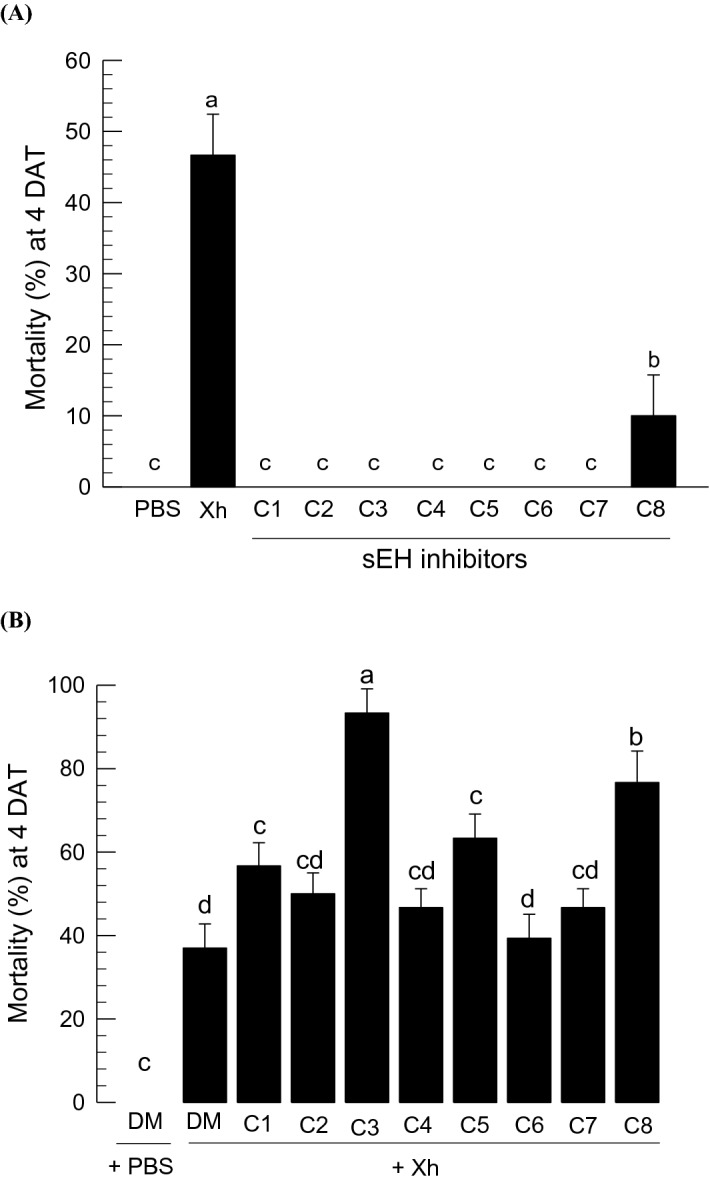
Enhanced pathogenicity of an entomopathogen, *X. hominickii* ANU101 against *S. exigua*, by addition of sEH inhibitor. The inhibitors include TPPU (ʽC1ʼ), PTUPB (ʽC2ʼ), AUDA (ʽC3ʼ), AEPU (ʽC4ʼ), *t*-AUCB (ʽC5ʼ), *t*-TUCB (ʽC6ʼ), *c*-TUCB (ʽC7ʼ), and AUCB (ʽC8ʼ). Control treatments used DMSO (‘DM’) or PBS used to dissolve inhibitors or bacteria, respectively. Inhibitor treatment used 0.1 μg per larva. Bacterial treatment used 10^4^ CFU. (**A**) Insecticidal activities of single treatments by injection to L5 larvae. (**B**) Insecticidal activities of mixed treatment with bacteria and inhibitor. Mortality was assessed 4 days after treatment. Each treatment used 10 larvae and was replicated three times. Different letters indicate significant differences among means at Type I error = 0.05 (LSD test).

Immunosuppressive activities of sEH inhibitors suggested that they might enhance pathogenicity of entomopathogens. *X. hominickii* was isolated from an entomopathogenic nematode, *Steinernema monticolum*, and exhibited significant entomopathogenicity. *X. hominickii* at a dose of 10^4^ cfu/larva exhibited about 42–45% mortality (Fig. 11A). Most sEH inhibitors (1 μg per larva) did not show any insecticidal activity except c-AUCB, which induced about 10% mortality. The bacterial pathogenicity was significantly enhanced when some of the urea derivatives were added in the bacterial challenge. AUDA, in particular, was most effective and enhanced the bacterial pathogenicity by more than 50% (Fig. 11B).

## Discussion

Eicosanoids and EpOMEs are derived from C20 arachidonic acid (AA) and LA, respectively. They are part of a large family of oxylipins. Eicosanoids are known to play crucial roles in mediating a number of physiological processes in insects^14^. In contrast, little is known about the physiological roles of EpOMEs in insects, although in humans, levels of C18 oxylipins are tightly regulated because alterations have been reported in a variety of disorders including painstates, hyperlipidaemia and diabetes^15^. This study detected two EpOMEs in *S. exigua* and assessed their immune-associated functions.

Using LC–MS/MS, two EpOMEs were detected in fat body of *S. exigua* larvae, with 12,13-EpOME (~ 7.41 nM) present at higher concentration than 9,10-EpOME (~ 3.17 nM). The levels detected in *S. exigua* are much lower than EpOME levels (50 ~ 100 nM) in human plasma^16^. The presence of EpOMEs in *S. exigua* was further supported by the prediction of EpOME synthases. Of 139 CYPs of *S. exigua, SE51385* was closely related to mammalian epoxygenases classified into CYP2C and CYP2J subfamilies^17^. Moreover, it was highly homologous (E value = 0.0) to a farnesoate epoxygenase (GenBank accession number: NP_001140197.1) of *Bombyx mori*, which was known to be CYP15C1 and functionally essential for juvenile hormone (JH) biosynthesis^18^. However, SE51385 was different to CYP15C1 in expression pattern. *SE51385* was expressed in all developmental stages and larval tissues while *CYP15C1* exhibited a specific expression in corpora allata, a JH source^18^. In the meantime, the JH epoxygenase of *S. exigua* may be SE50826, which is located in the EpOME synthase cluster and exhibits a higher similarity to farnesoate epoxygenase of *B. mori*. The similarity of SE51385 to other epoxygenases and a difference from farnesoate epoxygenase suggest its specific preference of catalytic substrates. To clarify its specific catalytic activity, recombinant SE51365 enzyme would be assessed in metabolites following incubation with methyl oleate, linoleate, and arachidonate along with methyl farnesoate. In addition to conserved domains shared with other CYPs, SE51385 is predicted to be located to membranes via GPI anchor. The membrane anchoring is crucial in transferring electrons from cytochrome P450 reductase during catalysis in mammals^19^. Another closely related CYP genes of *S. exigua* in the EpOME cluster was *Se-CYP18A1*, *Se-CYP18B1*, and *Se-CYP305B1*. These genes were identified as EET synthases (*SeEPX1-3*) of *S. exigua*^12^. In that study, these genes were expressed in all developmental stages and larval tissues, and RNAi of these genes led to suppression of cellular immune responses. Therefore, if these *EPX*s were responsible for EpOME synthesis, the RNAi treatments would not be expected to inhibit the immune response because EpOMEs suppress cellular immune responses. Thus, we conclude *Se-CYP18A1*, *Se-CYP18B1*, and *Se-CYP305B1* may be not associated with EpOME biosynthesis.

EpOMEs acted as anti-inflammatory mediators by suppressing both cellular and humoral immune responses in *S. exigua*. EpOME injection suppressed hemocyte behavior required for cellular immune responses and inhibited the induction of AMP gene expression. Expression of AMP genes are regulated by Toll/IMD signal pathways in *S. exigua*^20^. This suggests that EpOMEs may interrupt the Toll/IMD immune signals. In contrast, epoxy eicosanoids such as EETs increased the expression levels of AMPs in *S. exigua*^12^. This suggests there is a functional competition between these two types of oxylipin, in which EpOMEs may interfere with the functional mediation of EETs in AMP expression via Toll/IMD. Another possibility is a mediating role for DiHOMEs, the EpOME metabolites. In humans, EpOMEs and DiHOMEs are highly produced in neutrophils upon bacterial challenge^6,7^. Although the two oxylipins act as neutrophil chemotactic mediators^21^, only DiHOMEs suppress the excessive production of reactive oxygen species in neutrophils^10^. This anti-inflammatory role of DiHOMEs needs to be assessed in *S. exigua*.

RNAi treatment of *Se-sEH* expression led to significant immunosuppression of *S. exigua* larvae. Se-sEH was clustered with known mosquito orthologs and distinct from microsomal EHs including juvenile hormone epoxide hydrolases. sEH hydrolyzes epoxy fatty acids (EET or EpOME) to form dihydroxy fatty acids (DHET or DiHOME)^22^. Indeed, knocking sEH out, or inhibiting the enzyme with a selective inhibitor increased the ratios of EET/DHET and EpOME/DiHOME in mice^16^. Vertebrate sEHs are multidomain proteins containing N-terminal phosphatase and C-terminal α/β-hydrolase while bacteria, plants, and lower invertebrates including *Caenorhabdus elegans* possess only the C-terminal domain^23^. Interestingly, *C. elegans* genome encodes genes similar to N-terminal phosphatase of mammalian sEHs. This suggests that sEHs in vertebrates may be originated from gene fusion^24^. The first insect sEH was identified from a mosquito, *Anopheles gambiae*, and exhibited catalytic activity on 14,15-EET and 9,10-EpOME and was inhibited by a urea-based inhibitor, AUDA^25^. Another mosquito, *Culex quinquefasciatus*, also showed sEH activity in midgut, which was up-regulated in association with blood-feeding behavior and has been associated with microbial load in the midgut lumen^11^. In mammals, sEH has been regarded as a pro-inflammatory enzyme due to its activity in reducing anti-inflammatory EET levels^26^. In contrast, EETs appear to behave oppositely in insects. All four EETs act as pro-inflammatory signals by enhancing cellular and humoral immune responses in *S. exigua*^12^. In insect species, however, EpOMEs appear to behave in an anti-inflammatory manner as mentioned above. Se-sEH suppresses the levels of EpOMEs by its catalytic activity and may fail to inhibit immune responses. This may explain a role of Se-sEH as a pro-inflammatory agent in *S. exigua*. In response to immune challenge, the inducible expressions of EpOME synthase and Se-sEH genes suggest a tight regulation of EpOME level in time and locality at the infection site.

Inhibition of Se-sEH resulted in significant immunosuppression in *S. exigua*. RNAi treatment of *Se-sEH* expression suppressed cellular and humoral immune responses. Inhibitor screening against Se-sEH was performed with urea-based inhibitors and these experiments determined AUDA to be the most potent inhibitor. AUDA showed potent inhibitory activities against two different sEHs from *An. gambiae* and *Cx. quinquefastiatus*^11,25^. Ingestion of AUDA affected the growth of bacteria in the mosquito midgut, in which EpOMEs mimicked the effect of AUDA treatment^27^. This supports the pro-inflammatory role of sEH in *S. exigua* which probably functions by degrading EpOMEs.

The immunosuppressive effect of AUDA enhanced a bacterial pathogen, *X. hominickii*. Immune defenses of target insects typically attenuate bacterial pathogenicity^28^ and immunosuppression of target insects significantly increased the bacterial pathogenicity of *X. hominickii*^29^. These observations explain the role of AUDA in enhancing the bacterial pathogenicity because AUDA is likely to inhibit Se-sEH activity, which would increase EpOME levels thus driving suppression of immune responses. These results suggest that AUDA or its derivatives may be useful for developing novel insecticides to suppress insect immunity.

## Methods

### Insect rearing

Larvae of *S. exigua* were reared with an artificial diet^30^ under controlled conditions (25 °C, 16:8 h (L:D) photoperiod, and 60 ± 5% relative humidity). Adults were fed with 10% sucrose solution. Five larval instars (‘L1–L5’) were determined based on head capsule size^30^. Different larval tissues were isolated at 3 days, L5 (‘L5D3’) stage.

### Chemicals

9,10-EpOME and 12,13-EpOME were purchased from Cayman chemical company (Ann Arbor, MI, USA). Eight sEH inhibitors used in a previous study^11^ included TPPU (‘C1’) trifluoromethoxyphenyl-3-(1-propionylpiperidin-4-yl) urea, PTUPB (‘C2’) 4-(5-phenyl-3-{3-[3-(4-trifluoromethyl-phenyl)-ureido]-propyl}-pyrazol-1-yl)-benzenesulfonamide, AUDA (‘C3’) 12-(3-adamantan-1-yl-ureido) dodecanoic acid, AEPU (‘C4’) 1-adamantanyl-3-(5-(2-(2-ethoxyethoxy)ethoxy)pentyl))urea, *t*-AUCB (‘C5’) trans-4-[4-(3-adamantan-1-yl-ureido)-cyclohexyloxy]-benzoic acid, *t*-TUCB (‘C6’) trans-4-{4-[3-(4-trifluoromethoxyphenyl)-ureido]-cyclohexyloxy}-benzoic acid, *c*-TUCB (‘C7’) *cis*-4-{4-[3-(4-trifluoromethoxyphenyl)-ureido]-cyclohexyloxy}-benzoic acid, and *c*-AUCB (‘C8’) *cis*-4-[4-(3-adamantan-1-yl-ureido)-cyclohexyloxy]-benzoic acid. Dexamethasone (DEX), [(11β, 16α)-9-fluoro-11,17,21-trihydroxy-16-methylpregna-1,4-dione] and linoleic acid (LA) were purchased from Sigma-Aldrich Korea (Seoul, Korea).

### Bioinformatics to predict EpOME synthase and Se-sEH gene

To predict EpOME synthase of *S. exigua*, all CYPs were collected from *S. exigua* transcriptomes deposited to NCBI-GenBank (www.ncbi.nlm.nih.gov) and also several other CYPs were kindly provided by Professor Salvador Herrero (Department of Genetics, University of Valencia, Spain). After aligning all candidate sequences using ClustalW of MegAlign (DNASTAR Version 7.0, Madison, WI, USA), CYP genes were collected. Their GenBank accession numbers are described in Supplementary Table S1. *S. exigua* CYP genes were compared with EpOME synthases of human^31^ and mice^32^ that were collected from NCBI-GenBank (Supplementary Table S1). The predicted amino acid sequences of sEHs (Supplementary Table S2) were aligned using Clustal W. The phylogenetic trees for CYPs and sEHs were constructed with Neighbor-joining method and Poisson correction model (1000 bootstrap repetitions to support branching clusters) using MEGA 7.0 (www.megasoftware.net). A cDNA sequence (GenBank: ETN62858.1) of *Anopheles darlingi* EH was used to probe an *S. exigua* hemocyte transcriptome (SRX259774). Four partial contigs were matched (E value < 10^–10^) and assembled to be a full sequence. Conserved domains of Se-sEH was predicted using NCBI Conserved Domain Database (www.ncbi.nlm.nih.gov/cdd). Membrane anchoring was predicted by using a GPI modification site prediction engine supported by Expasy bioinformatics tool (https://www.expasy.org/proteomics). Swiss-PDB Viewer (https://spdbv.vital-it.ch/) and UCSF Chimera (https://www.cgl.ucsf.edu/chimera/) were used for protein motif analysis. The model of protein was created by SWISS-MODEL website (https://swissmodel.expasy.org/) using amino acids sequences. Resulted model was submitted to Chimera V1.14 software to construct 3D dimensional structure of proteins.

### RNA extraction, RT-PCR, and qPCR

Total RNAs were extracted from whole body, different tissues, and various developmental stages (100 eggs, 20 young larvae (L1–L3), three L4 larvae, one L5 larva, one pupa, and one adult for each extraction) of *S. exigua*. RNA extraction used Trizol reagent (Invitrogen, Carlsbad, CA, USA) according to the manufacturer’s instruction. The extracted RNA was treated with RNase-free DNase (Bioneer, Seoul, Korea) to remove any genomic DNA contamination and used for cDNA synthesis (0.5 μg RNA per reaction) using RT-premix (Intron Biotechnology, Seoul, Korea). The synthesized cDNA was used as template for PCR amplification with *Se-sEH*-specific forward and reverse primers (Supplementary Table S3). Expression of ribosomal protein L32, *RL32*, was assessed with its gene-specific primers to determine cDNA integrity. After an initial denaturation at 95 °C for 1 min, PCR was performed with 35 cycles of denaturation at 95 °C for 1 min, annealing at 52 °C for 1 min, and extension at 72 °C for 1 min. PCR reaction was terminated with a final extension step at 72 °C for 10 min. PCR products were separated on 1% agarose gel by electrophoresis. Quantitative PCR (qPCR) was performed using SYBR Green Real time PCR master mixture (Toyobo, Osaka, Japan) on a StepOne real time PCR system (Applied Biosystems, Foster, CA, USA) according to the manufacturer's instruction. The reaction mixture (20 μL) contained 25 pmol of primers used in RT-PCR as described above and 80 ng of cDNA template. After activating Hotstart Taq DNA polymerase at 94 °C for 5 min, the reaction was amplified with 40 cycles of denaturation at 94 °C for 30 s, annealing at 52 °C for 30 s, and extension at 72 °C for 30 s. Fluorescence values were measured and amplification plots were generated in real time by Applied Biosystems Manager. Expression levels of *RL32* were used as internal control in each qPCR reaction. Fluorescence emitted from the newly synthesized PCR products at every cycle was monitored to quantify PCR products. Melting curves of PCR products were analyzed to confirm single product. Each treatment was replicated with three independent biological sample preparations. Quantitative analysis of gene expression was performed using a comparative cycle threshold (CT) method^33^.

### Silencing *Se-sEH* expression by RNA interference (RNAi)

RNAi was performed using dsRNA prepared with Megascript RNAi Kit (Ambion, Austin, TX, USA) according to the manufacturer’s instruction. A partial *Se-sEH* (354 bp) and *SE51385* (400 bp) were amplified with gene-specific primers containing T7 promoter sequence at the 5′ end (Supplementary Table S3). dsRNAs (‘ds-sEHʼ and ‘ds-SE51385’) were synthesized at 37 °C for 4 h and then left at 70 °C for 5 min to inactivate T7 RNA polymerase. As control dsRNA (‘dsCON’), a 520 bp fragment of green fluorescent protein (*GFP*) was synthesized. One µg of dsRNA was injected into one day old L4 (L4D1) larvae using a microsyringe (Hamilton, Reno, NV, USA). RNAi efficiency was determined by qPCR as described above at 24, 48, 72 and 96 h PI. For each treatment, 10 larvae were used. Each treatment was replicated three times.

### Hemocyte-spreading assay using in situ fluorescence assay

Hemolymph (∼200 μL) from five L5D3 larvae was collected in 800 μL of cold anticoagulant buffer (186 mM NaCl, 17 mM EDTA, 41 mM citric acid, pH 4.5) and incubated on ice for 30 min. After incubation, hemolymph was separated into hemocytes and plasma by centrifugation at 800 × *g* for 5 min at 4 °C. The pellet was re-suspended in 500 μL of filter-sterilized TC-100 insect cell culture medium (Welgen, Daegu, Korea). On a glass coverslip, 10 μL of hemocyte suspension was applied and the attaching hemocytes were fixed with 4% paraformaldehyde for 10 min at 25 °C. After washing three times with filter-sterilized 100 mM phosphate-buffered saline (PBS, pH 7.4), hemocytes were then permeabilized with 0.2% Triton-X in PBS for 2 min at 25 °C. The treated hemocytes were incubated with 10% bovine serum albumin (BSA) for 10 min at 25 °C and subsequently with fluorescein isothiocyanate (FITC)-tagged phalloidin (Thermo Fisher Scientific Korea, Seoul, Korea) in PBS for 60 min. Hemocyte nuclei were then stained with 4′,6-diamidino-2-phenylindole (DAPI, 1 μg/mL) (Thermo Fisher Scientific Korea). Hemocyte-spreading was determined based on extension of F-actin beyond the cell boundary of hemocytes under a fluorescence microscope (DM2500, Leica, Wetzlar, Germany) at 400 × magnification.

### Hemocyte nodulation assay

Hemocytic nodules are formed as a type of cellular immune response of *S. exigua* after challenging with bacteria^34^. ds-sEH was injected into each L4 larva at a dose of 1 μg. Larvae were then reared with diet at 25 °C for 48 h. At 48 h after dsRNA treatment, 5 × 10^5^ cells of heat-killed (95 °C for 10 min) *E. coli* was injected into each larva and incubated at 25 °C for 8 h. After dissection under a stereomicroscope (Stemi SV11, Zeiss, Jena, Germany), the number of nodules was counted at 60 × magnification. Nodules at gut, trachea, and fat body were observed. Each treatment was replicated with 10 larvae.

### LC–MS/MS analysis

LC–MS/MS was performed using a QTrap 4500 (AB Sciex, Framingham, MA, USA) equipped with auto-sampler, a binary pump, and column oven. The analytical column was an Osaka Soda (Osaka, Japan) C18 column (2.1 mm × 150 mm, 2.7 μm) maintained at 40 °C. The mobile phases consisted of 0.1% formic acid in water (A) and 0.1% formic acid in acetonitrile (B). The linear gradient was as follows: 30% B at 0 min, 30% B at 2 min, 65% B at 12 min, 95% B at 12.5 min, 95% B at 25.0 min, 30% B at 28.0 min, and 30% B at 30 min. The flow rate was 0.40 mL/min. The auto sampler was set at 5 °C and the injection volume was 10 μL. LC–MS/MS was equipped with electrospray ionization (ESI) source. ESI was performed in negative ion mode. After optimization, the source parameters used were as follows: source temperature at 600 °C, curtain gas flow rate at 32 L/min, ion source gas flow rate at 60 L/min, and the spray voltage adjusted to − 4000 V. Analyses were performed in multiple reaction monitoring detection mode using nitrogen as collision gas. Peak detection, integration, and quantitative analysis were done using MassView1.1 software (AB Sciex, Seoul, Korea).

### Analysis of EpOMEs on cellular immune responses

Different doses (10^–10^ ~ 10^–6^ M) of 9,10-EpOME or 12,13-EpOME were injected to L5D3 larvae of *S. exigua*. Hemocyte-spreading was determined based on extension of F-actin out of the cell boundary of the hemocyte. For nodulation assay, 9,10-EpOME or 12,13-EpOME was injected to L5D3 larvae at a dose of 100 ng per larva along with 5 × 10^5^ cells of *E. coli*. After 8 h incubation, nodules were counted as described above. Each treatment was performed in triplicate using ten insects per replicate.

### Analysis of EpOMEs on humoral immune responses

L5D3 larvae were injected with 9,10-EpOME or 12,13-EpOME (100 ng per larva) along with 5 × 10^5^ cells of *E. coli*. After 8 h incubation, fat body tissues were isolated and used for RNA extractions as described above. For each replication, 10 larvae were used for collecting tissues and subsequent RNA extraction. Each treatment used three independent sample collections. Ten AMPs were assessed for their expression levels by RT-qPCR using gene-specific primers (Table S3).

### Screening of sEH inhibitors against immune responses

Hemocyte-spreading and nodule formation were assessed to screen efficacies of sEH inhibitors. L5D3 were injected in one μL volume containing either the inhibitors at 0.1 µg/larva or DMSO as the control. After 8 h incubation, hemolymph was collected and hemocyte spreading was evaluated. The test larvae were also subjected to nodule count by dissection. The fat body samples collected after dissection were used to assay AMP expression as described above. Each treatment was performed in triplicate using ten insects per replicate.

### Screening of sEH inhibitors against bacterial pathogenicity

For bacterial pathogenicity assessment, an entomopathogenic bacterium (*Xenorahbdus hominickii* ANU101) was cultured in tryptic soy broth medium (TSB) (Difco, Detroit, MI, USA) at 28 °C. After washing the cultured bacteria three times with PBS by centrifugation at 4000 × *g* at 4 °C, 10^4^ colony-forming unit (cfu) of bacteria were injected into each L5D3 larva along with sEH inhibitors or DMSO. Mortality was assessed at 4 days PI. Each treatment was performed in triplicate using ten insects per replicate.

### Data analysis

All studies were performed with three independent biological replicates. Results were plotted using Sigma plot 10.0. Means were compared by least squared difference (LSD) test of one-way analysis of variance (ANOVA) using PROC GLM of SAS program and discriminated at Type I error = 0.05.

## Supplementary information

Supplementary Information.
