# Supplementary material for: EpOMEs act as immune suppressors in a lepidopteran insect, Spodoptera exigua
Source: Sci Rep. 2020 Nov 19;10:20183. doi: 10.1038/s41598-020-77325-2 (PMC7677322; doi:10.1038/s41598-020-77325-2)
Supplement: Supplementary file 1 — Supplementary Information. [file 41598_2020_77325_MOESM1_ESM.pdf]

Supplementary data

EpOMEs act as immune suppressors in a lepidopteran insect, *Spodoptera exigua*

Mohammad Vatanparast[1], Shabbir Ahmed[1], Dong-Hee Lee[2], Sung Hee Hwang[3], Bruce Hammock[3] & Yonggyun Kim[1*]

[1]Department of Plant Medicals, Andong National University, Andong 37629, Korea
[2]Industry Academy Cooperation Foundation, Andong National University, Andong 36729, Korea
[3]Department of Entomology and Nematology, UC Davis Comprehensive Cancer Center, University of California, Davis, CA, USA

[*]Corresponding author: Email) hosanna@anu.ac.kr

Table S1. GenBank accession numbers and abbreviations used for phylogenetic analysis of EpOME synthases

Table S2. GenBank accession numbers and abbreviations used for phylogenetic analysis of sEH

Table S3. Primer sequences used in this study

Fig. S1. Chromatograms of LC-MS/MS to measure EpOMEs extracted from fat body samples (‘IMMUNE1-3’) of *S. exigua* larvae (A) 9,10-EpOME measurement using ion peaks at 295.0/195.0. (B) 9,10-EpOME measurement using ion peaks at 295.0/277.1. (C) 12,13-EpOME measurement using ion peaks at 294.9/78.9. Each EpOME analysis used five different doses of standard.

Fig. S2. Sequence alignment of a putative EpOME synthase (‘SE51385’) encoded in *S. exigua* with two mammalian orthologs: CYP2J6 of *Mus musculus* (‘MMCYP2J6’) and CYP2J2 of *Homo sapiens* (‘HSCYP2J2’). Conserved domains are denoted with colored shades at proline-rich, four substrate recognition sites (‘SRS-1 ~ SRS-4’), and heme-binding regions.

Fig. S3. Sequence alignment of a putative soluble epoxide hydrolase ('Sexi') encoded in *S. exigua* with four other insect orthologs of *Aedes aegypti* ('Aaeg'), *Bombyx mori* ('Bmor'), *P. xylostella* ('Pxyl'), and *Helicoverpa armigera* ('Har'). Conserved domains containing catalytic residues are denoted with colored shades. The three putative active site residues (D/H/D) are marked in red. Tyrosines are shown in blue. The conserved HGXP and GXSmXS/T motifs are highlighted in yellow. Sequence alignment used Clustal W program of MegAlign (DNASTAR, Version 7.0)

Fig. S4. Chemical structure of urea-based derivatives as putative sEH inhibitors

Fig. S5. Effect of soluble epoxide hydrolase (sEH) inhibitors on expression of seven antimicrobial peptides (AMPs). The inhibitors include TPPU ('C1'), PTUPB ('C2'), AUDA ('C3'), AEPU ('C4'), *t*-AUCB ('C5'), *t*-TUCB ('C6'), *c*-TUCB ('C7'), and AUCB ('C8'). Control treatment ('CON') was vehicle solvent (DMSO). (A) Expression of seven AMP genes (RT-PCR). The AMP genes include *attacin 1* ('Att1'), *attacin 2* ('Att2'), *cecropin* ('Cec'), *defensin* ('Def'), *gallerimycin* ('Gal'), *transferrin 1* ('Tf1'), and *transferrin 2* ('Tf2'). Each inhibitor was injected at a dose of 0.1 µg/larva along with bacteria (*E. coli*, 5×10^5 cells/larva). (A-G) Quantitative analysis of the AMP genes at 8 h PI using RT-qPCR. Each treatment was independently replicated three times. Different letters indicate significant differences among means at Type I error = 0.05 (LSD test).

Fig. S6. Uncropped gel pictures in this study. (A) Fig. 5A (B) Fig. 8A

Table S1. GenBank accession numbers and abbreviations used for phylogenetic analysis of EpOME synthases

Species	Abbreviation	Gene	Accession number*
Spodoptera exigua	SE 44208	Cytochrome_P450	c17986.44208
Spodoptera exigua	SE CYP4G75	Cytochrome_P450_CYP4G75	ASO98035.1
Spodoptera exigua	SE CYP4G74	Cytochrome_P450_CYP4G74	ASO98034.1
Spodoptera exigua	SE CYP340AB1	Cytochrome_P450_CYP340AB1	ASO98033.1
Spodoptera exigua	SE CYP340AA1	Cytochrome_P450_CYP340AA1	ASO98032.1
Spodoptera exigua	SE CYP307A2	Cytochrome_P450_CYP307A2	ASO98031.1
Spodoptera exigua	SE ABL63810	Cytochrome_P450	ABL63810.1
Spodoptera exigua	SE ACL77781	Cytochrome_P450	ACL77781.1
Spodoptera exigua	SE 47469	Cytochrome_P450	C17986.47469
Spodoptera exigua	SE 49335	Cytochrome_P450	c17986.49335
Spodoptera exigua	SE ATP15899	Cytochrome_P450	ATP15899.1
Spodoptera exigua	SE 14865	Cytochrome_P450	c17986.14865
Spodoptera exigua	SE CYP9A21v3	Cytochrome_P450_CYP9A21v3	AYA28075.1
Spodoptera exigua	SE CYP9A21v2	Cytochrome_P450_CYP9A21v2	AYA28074.1
Spodoptera exigua	SE 50826	Cytochrome_P450	c17986.50826
Spodoptera exigua	SE c37730.0	Cytochrome_P450	c37730.0
Spodoptera exigua	SE NADPH	NADPH_cytochrome_P450_reductase	ADX95746.1
Spodoptera exigua	SE CYP9A	Cytochrome_P450_9A9	BAG71410.1
Spodoptera exigua	SE CYP428A1	Cytochrome_P450_CYP428A1	ASO98058.1
Spodoptera exigua	SE CYP339A1	Cytochrome_P450_CYP339A1	ASO98057.1
Spodoptera exigua	SE 75876	Cytochrome_P450	c17986.75876
Spodoptera exigua	SE CYP333B4	Cytochrome_P450_CYP333B4	ASO98055.1
Spodoptera exigua	SE CYP333A12	Cytochrome_P450_CYP333A12	ASO98054.1
Spodoptera exigua	SE CYP315A1	Cytochrome_P450_CYP315A1	ASO98053.1
Spodoptera exigua	SE CYP314A1	Cytochrome_P450_ CYP314A1	ASO98052.1
Spodoptera exigua	SE CYP302A1	Cytochrome_P450_CYP302A1	ASO98051.1
Spodoptera exigua	SE CYP301B1	Cytochrome_P450_CYP301B1	ASO98050.1
Spodoptera exigua	SE CYP301A1	Cytochrome_P450_CYP301A1	ASO98049.1
Spodoptera exigua	SE CYP367B1	Cytochrome_P450_CYP367B1	ASO98048.1
Spodoptera exigua	SE CYP367A1	Cytochrome_P450_CYP367A1	ASO98047.1
Spodoptera exigua	SE CYP341B26	Cytochrome_P450_CYP341B26	ASO98046.1
Spodoptera exigua	SE CYP341A11	Cytochrome_P450_CYP341A11	ASO98045.1
Spodoptera exigua	SE CYP4M18	Cytochrome_P450_CYP4M18	ASO98044.1
Spodoptera exigua	SE CYP4M17	Cytochrome_P450_CYP4M17	ASO98043.1
Spodoptera exigua	SE CYP4M15	Cytochrome_P450_CYP4M15	ASO98042.1
Spodoptera exigua	SE CYP4M14	Cytochrome_P450_CYP4M14	ASO98041.1
Spodoptera exigua	SE CYP4L15	Cytochrome_P450_CYP4L15	ASO98040.1
Spodoptera exigua	SE CYP4L9	Cytochrome_P450_CYP4L9	ASO98039.1
Spodoptera exigua	SE 53716	Cytochrome_P450	c17986.53716
Spodoptera exigua	SE 20762	Cytochrome_P450	c17986.20762
Spodoptera exigua	SE CYP4S8	Cytochrome_P450_CYP4S8	ASO98036.1
Spodoptera exigua	SE CYP306A1	Cytochrome_P450_CYP306A1	ASO98030.1
Spodoptera exigua	SE CYP305B1	Cytochrome_P450_CYP305B1	ASO98029.1
Spodoptera exigua	SE CYP304F1	Cytochrome_P450_CYP304F1	ASO98028.1
Spodoptera exigua	SE CYP18B1	Cytochrome_P450_CYP18B1	ASO98027.1
Spodoptera exigua	SE CYP18A1	Cytochrome_P450_CYP18A1	ASO98026.1
Spodoptera exigua	SE CYP354A14	Cytochrome_P450_CYP354A14	ASO98025.1
Spodoptera exigua	SE CYP338A1	Cytochrome_P450_CYP338A1	ASO98024.1
Spodoptera exigua	SE CYP337B5	Cytochrome_P450_CYP337B5	ASO98023.1
Spodoptera exigua	SE 43305	Cytochrome_P450	c17986.43305
Spodoptera exigua	SE CYP324A	Cytochrome_P450_CYP324A1	ASO98021.1
Spodoptera exigua	SE CYP321B4	Cytochrome_P450_CYP321B4	ASO98020.1
Spodoptera exigua	SE CYP321B1	Cytochrome_P450_CYP321B1	ASO98019.1
Spodoptera exigua	SE CYP321A16	Cytochrome_P450_CYP321A16	ASO98018.1
Spodoptera exigua	SE 31299	Cytochrome_P450	c17986.31299
Spodoptera exigua	SE 66279	Cytochrome_P450	c17986.66279
Spodoptera exigua	SE CYP9A98	Cytochrome_P450_CYP9A98	ASO98015.1
Spodoptera exigua	SE CYP9A27	Cytochrome_P450_CYP9A27	ASO98014.1
Spodoptera exigua	SE 76146	Cytochrome_P450	c17986.76146
Spodoptera exigua	SE 47400	Cytochrome_P450	c17986.47400
Spodoptera exigua	SE 53088	Cytochrome_P450	c17986.53088
Spodoptera exigua	SE 46792	Cytochrome_P450	c17986.46792

Spodoptera exigua	SE CYP6AE74	Cytochrome_P450_CYP6AE74	ASO98009.1
Spodoptera exigua	SE CYP6AE70	Cytochrome_P450_CYP6AE70	ASO98008.1
Spodoptera exigua	SE 83845	Cytochrome_P450	c17986.83845
Spodoptera exigua	SE CYP6AE47	Cytochrome_P450_CYP6AE47	ASO98006.1
Spodoptera exigua	SE 28012	Cytochrome_P450_CYP6AE10	c17986.28012
Spodoptera exigua	SE CYP6B68	Cytochrome_P450_CYP6B68	ASO98004.1
Spodoptera exigua	SE CYP6B50	Cytochrome_P450_CYP6B50	ASO98003.1
Spodoptera exigua	SE 44858	Cytochrome_P450	c17986.44858
Spodoptera exigua	SE 44378	Cytochrome_P450	c17986.44378
Spodoptera exigua	SE CYP6AB61	Cytochrome_P450_CYP6AB61	ASO98000.1
Spodoptera exigua	SE CYP6AB31	Cytochrome_P450_CYP6AB31	ASO97999.1
Spodoptera exigua	SE CYP6AB14	Cytochrome_P450_CYP6AB14	ASO97998.1
Spodoptera exigua	SE CYP6AB12	Cytochrome_P450_CYP6AB12	ASO97997.1
Spodoptera exigua	SE CYP366A1	Cytochrome_P450_CYP366A1	ASN63844.1
Spodoptera exigua	SE CYP341B27	Cytochrome_P450_CYP341B27	ASN63843.1
Spodoptera exigua	SE CYP340L1	Cytochrome_P450_CYP340L1	ASN63842.1
Spodoptera exigua	SE CYP340K4	Cytochrome_P450_CYP340K4	ASN63841.1
Spodoptera exigua	SE 43989	Cytochrome_P450	c17986.43989
Spodoptera exigua	SE 77520	Cytochrome_P450	c17986.77520
Spodoptera exigua	SE 70841	Cytochrome_P450	c17986.70841
Spodoptera exigua	SE 84605	Cytochrome_P450	c17986.84605
Spodoptera exigua	SE 50006	Cytochrome_P450	c17986.50006
Spodoptera exigua	SE 36527	Cytochrome_P450	c17986.36527
Spodoptera exigua	SE 46274	Cytochrome_P450	c17986.46274
Spodoptera exigua	SE 49762	Cytochrome_P450	c17986.49762
Spodoptera exigua	SE 65099	Cytochrome_P450	c17986.65099
Spodoptera exigua	SE 20346	Cytochrome_P450	c17986.20346
Spodoptera exigua	SE 14880	Cytochrome_P450	c17986.14880
Spodoptera exigua	SE 84271	Cytochrome_P450	c17986.84271
Spodoptera exigua	SE 46030	Cytochrome_P450	c17986.46030
Spodoptera exigua	SE 26529	Cytochrome_P450	c17986.26529
Spodoptera exigua	SE 46406	Cytochrome_P450	c17986.46406
Spodoptera exigua	SE 41099	Cytochrome_P450	c17986.41099
Spodoptera exigua	SE 30431	Cytochrome_P450	c17986.30431
Spodoptera exigua	SE 35312	Cytochrome_P450	c17986.35312
Spodoptera exigua	SE 5014	Cytochrome_P450	c17986.5014
Spodoptera exigua	SE 47554	Cytochrome_P450	c17986.47554
Spodoptera exigua	SE 73734	Cytochrome_P450	c17986.73734
Spodoptera exigua	SE 48426	Cytochrome_P450	c17986.48426
Spodoptera exigua	SE 83896	Cytochrome_P450	c17986.83896
Spodoptera exigua	SE 10755	Cytochrome_P450	c17986.10755
Spodoptera exigua	SE 51385	Cytochrome_P450	c17986.51385
Spodoptera exigua	SE 85599	Cytochrome_P450	c17986.85599
Spodoptera exigua	SE 57908	Cytochrome_P450	c17986.57908
Spodoptera exigua	SE 47083	Cytochrome_P450	c17986.47083
Spodoptera exigua	SE 44691	Cytochrome_P450	c17986.44691
Spodoptera exigua	SE 50470	Cytochrome_P450	c17986.50470
Spodoptera exigua	SE 68152	Cytochrome_P450	c17986.68152
Spodoptera exigua	SE 6184	Cytochrome_P450	c17986.6184
Spodoptera exigua	SE 23074	Cytochrome_P450	c17986.23074
Spodoptera exigua	SE 50723	Cytochrome_P450	c17986.50723
Spodoptera exigua	SE 49404	Cytochrome_P450	c17986.49404
Spodoptera exigua	SE 81107	Cytochrome_P450	c17986.81107
Spodoptera exigua	SE 51663	Cytochrome_P450	c17986.51663
Spodoptera exigua	SE 45000	Cytochrome_P450	c17986.45000
Spodoptera exigua	SE 53726	Cytochrome_P450	c17986.53726
Spodoptera exigua	SE 82891	Cytochrome_P450	c17986.82891
Spodoptera exigua	SE 46678	Cytochrome_P450	c17986.46678
Spodoptera exigua	SE 48506	Cytochrome_P450	c17986.48506
Spodoptera exigua	Se 44096	Cytochrome_P450	c17986.44096
Spodoptera exigua	SE 16626	Cytochrome_P450	c17986.16626
Spodoptera exigua	SE 41866	Cytochrome_P450	c17986.41866
Spodoptera exigua	SE 84009	Cytochrome_P450	c17986.84009
Spodoptera exigua	SE 49141	Cytochrome_P450	c17986.49141
Spodoptera exigua	SE 44069	Cytochrome_P450	c17986.44069
Spodoptera exigua	SE 40748	Cytochrome_P450	c17986.40748

Spodoptera exigua	SE 44116	Cytochrome_P450	c17986.44116
Spodoptera exigua	SE 29201	Cytochrome_P450	c17986.29201
Spodoptera exigua	SE 47327	Cytochrome_P450	c17986.47327
Spodoptera exigua	SE c19332.0	Cytochrome_P450	c19332.0
Spodoptera exigua	SE c15761.0	Cytochrome_P450	c15761.0
Spodoptera exigua	SE c8847.0	Cytochrome_P450	c8847.0
Spodoptera exigua	SE c27582.0	Cytochrome_P450	c27582.0
Spodoptera exigua	SE c8156.0	Cytochrome_P450	c8156.0
Spodoptera exigua	SE c43994.0	Cytochrome_P450	c43994.0
Spodoptera exigua	SE c15696.0	Cytochrome_P450	c15696.0
Spodoptera exigua	SE c23145.0	Cytochrome_P450	c23145.0
Spodoptera exigua	SE c19068.0	Cytochrome_P450	c19068.0
Homo sapiens	HS CYP2C8	Cytochrome_P450	1PQ2_A
Homo sapiens	HS CYP2J2	Cytochrome_P450	NP_000766.2
Homo sapiens	HS CYP3A4	Cytochrome_P450	NP_059488.2
Homo sapiens	HS CYP1A2	Cytochrome_P450	NP_000752.2
Homo sapiens	HS CYP2E1	Cytochrome_P450	NP_000764.1
Homo sapiens	HS CYP2C9	Cytochrome_P450	NP_000762.2
Homo sapiens	HS CYP2C19	Cytochrome_P450	NP_000760.1
Mus musculus	MM CYP2J6	Cytochrome_P450	NP_034138.3
Mus musculus	MM CYP1A2	Cytochrome_P450	NP_034123.1
Mus musculus	MM CYP2J5	Cytochrome_P450	NP_034137.1
Mus musculus	MM CYP2E1	Cytochrome_P450	NP_067257.1
Mus musculus	MM CYP2J9	Cytochrome_P450	NP_083255.1
Rattus norvegicus	RN CYP2J3	Cytochrome_P450	NP_786942.1

Table S2. GenBank accession numbers and abbreviations used for phylogenetic analysis of soluble epoxide hydrolases

**Species**	**Abbreviation**	**Gene**	**Accession number***
Acanthaster planci	Ap	Epoxide hydrolase 4-like	XP_022103784.1
Aedes aegypti	Aaeg	Epoxide hydrolase 4	AAEL006354
Aedes aegypti	Aaeg PA	Soluble epoxide hydrolase 4	AAEL006354
Aedes aegypti	Aaeg1	Soluble epoxide hydrolase 1	XP_001651935.1
Aedes aegypti	Aaeg PB	Soluble epoxide hydrolase	EAT42095.1
Amphimedon queenslandica	Aq	Epoxide hydrolase 4	XP_011405812.1
Anopheles gambiae	Agam1	Microsomal epoxide hydrolase	EAA10122.2
Anopheles gambiae	Agam2	Microsomal epoxide hydrolase	EAL40266.2
Anopheles gambiae	Agam3	Microsomal epoxide hydrolase	EAL40265.1
Biomphalaria glabrata	Bg	Epoxide hydrolase 1-like	XP_013068873.1
Bombyx mori	Bmor	Epoxide hydrolase 4 isoform X1	XP_004927786.1
Bombyx mori	Bmor X1	Soluble epoxide hydrolase 4 isoform X1	XP_004927786.1
Bombyx mori	Bm	Soluble epoxide hydrolase 4 isoform X1	XP_004927786.1
Bombyx mori	Bmor X2	Soluble epoxide hydrolase 4 isoform X2	XP_021204834.1
Bombyx mori	Bmor	Microsomal epoxide hydrolase	BAH97088.1
Caenorhabditis elegans	Cele	Soluble epoxide hydrolase 1	CCD61875.1
Caenorhabditis elegans	Ce	Epoxide hydrolase 1	NP_497268.1
Callorhinchus milii	Cm	Epoxide hydrolase 4-like	XP_007908548.1
Capitella teleta	Ct	Epoxide hydrolase	ELT96409.1
Chiroxiphia lanceolata	Cl	Epoxide hydrolase 4	XP_032552002.1
Drosophila melanogaster	Dmel	Microsomal epoxide hydrolase	BAD04047.1
Erpetoichthys calabaricus	Eca	Epoxide hydrolase 4	XP_028667268.1
Escherichia coli	Ec	Epoxide hydrolase	MXG36127.1
Gallus	Ggal	Soluble epoxide hydrolase	AAZ38461.1
Helicoverpa armigera	Har	Epoxide hydrolase 3-like	XP_021183905.1
Homo sapiens	Hsap, Hs	Soluble epoxide hydrolase 4	NP_775838.3
Homo sapiens	Hsap	Microsomal epoxide hydrolase	AAC41694.1
Lacerta agilis	La	Epoxide hydrolase 4	XP_033007499.1
Macrostomum lignano	Ml	Epoxide hydrolase	PAA58038.1
Microcaecilia unicolor	Mu	Epoxide hydrolase 3	XP_030053048.1
Mus musculus domesticus	Mmus	Soluble epoxide hydrolase	AAA37555.1
Petromyzon marinus	Pm	Epoxide hydrolase 4-like isoform X2	XP_032828833.1
Plutella xylostella	Pxyl	Epoxide hydrolase 3-like	XP_011556182.1
Pocillopora damicornis	Pd	Epoxide hydrolase 4-like	XP_027049700.1
Rattus norvegicus	Rnor	Soluble epoxide hydrolase	CAA46211.1
Rattus norvegicus	Rnor	Microsomal epoxide hydrolase	AAA41585.1
Spodoptera exigua	Se-JHEH	Juvenile hormone epoxide hydrolase	ABD85119.1
Spodoptera litura	Slit	Soluble epoxide hydrolase 3-like	XP_022829648.1
Strongylocentrotus purpuratus	Spur	Soluble epoxide hydrolase-like protein 1	NP_001121538.1
Thermococcus barophilus	Tb	Epoxide hydrolase	WP_056933353.1

Table S3. Primer sequences used in this study

Primer	Use	Orientation	Sequence (5′ - 3′)	Annealing temperature (°C)	Amplicon (bp)
Se_sEH	RT-PCR	Forward<br>Reverse	TTCAGATGGAGACGGGAGAT<br>ACAGCCTCGAACATCGTATTG	52	308
qSe_sEH	qPCR	Forward<br>Reverse	TATGCCTCGGTTCTACCAAAC<br>ACTGGTTTAGCTCCACCATAAG	52	104
RL32	qRT-PCR	Forward<br>Reverse	ATGCCCAACATTGGTTACGG<br>TTCGTTCTCCTGGCTGCGGA	52	270
dsCON	RNAi	Forward<br>Reverse	CCCACTAGTGTCCTCATCACCTCCTCAAAC<br>CCCAAGCTTCAGAGTCACCGTTGCAAGTA	52	520
T7Se_sEH	RNAi	Forward<br>Reverse	TAATACGACTCACTATAGGGAGA TTCAGATGGAGACGGGAGAT<br>TAATACGACTCACTATAGGGAGAACAGGAACAA CTCCATCCTTG	54	354
Gloverin	qPCR	Forward<br>Reverse	CGTGGACATCTTCAGGGCC<br>GTCGTGTTCAATGCCACCG	54	277
Lysozyme	qPCR	Forward<br>Reverse	ATGCAAAAGCTAACGGTTTTC<br>GATTCTTCCATCCATACCAG	54	385
Cecropin	qPCR	Forward<br>Reverse	ATCGTTTAGCTTCGTGTTCG<br>CTTTCTTTTACCACACGGTT	54	331
Apolipophorin III	qPCR	Forward<br>Reverse	AGTGTCGCCAAGTTGTTCGTG<br>CTCCTGCGCGGTGTTCTGCA	52	420
Attacin1	qPCR	Forward<br>Reverse	GCTTTCCTCTCCAGGAATATG<br>CCTTAGAGTAAATCCAGTGG	52	276
Attacin2	qPCR	Forward<br>Reverse	TCCCGAATGTGCCCAACTTC<br>GAAAGATCTGCCGAAAGTAAG	52	254
Defensin	qPCR	Forward<br>Reverse	ATGGGTGTTAAGGTAATAAATGTG<br>GCAACTACATGTATGACTAACGC	52	303
Gallerimycin	qPCR	Forward<br>Reverse	TCAGTCATGAAAGCTTGCGTA<br>TCGCACACATTGGCATCCATTC	52	222
Transferrin1	qPCR	Forward<br>Reverse	GTCCCTCTCTGTCCTGAAGG<br>CAGAAACACGAAGAAAGATGG	52	370
Transferrin2	qPCR	Forward<br>Reverse	GATGTTCTGGCGCAGCTGTC<br>CCGGCTGAACGCAAACACAG	52	288
SE51385	RT-PCR	Forward<br>Reverse	CCCATCATAGCACTCTCTGTTC<br>GGTCCATCGTTGAAGACTATCC	52	353
qSE51385	qPCR	Forward<br>Reverse	TTGTATCGGCGAAGGATTGG<br>AGTCTCGCCATCTGGTATCT	52	94
T7SE51385	qPCR	Forward<br>Reverse	TAATACGACTCACTATAGGGAGACCCATCATAG CACTCTCTGTTC<br>TAATACGACTCACTATAGGGAGAGGTCCATCGT TGAAGACTATCC	52	400

(A)

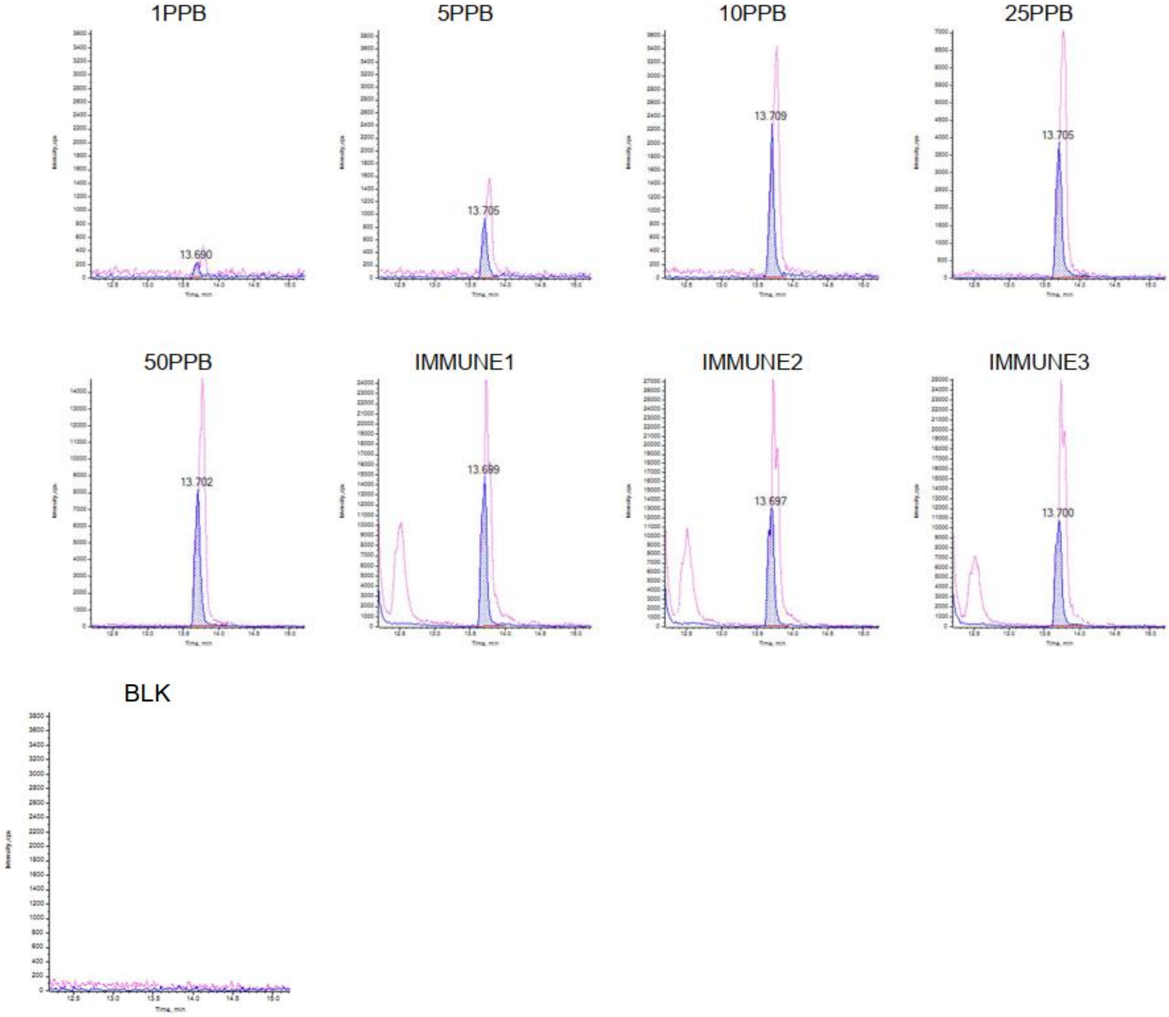
1PPB
13.690
5PPB
13.705
10PPB
13.709
25PPB
13.705
50PPB
13.702
IMMUNE1
13.699
IMMUNE2
13.697
IMMUNE3
13.700
BLK

(B)

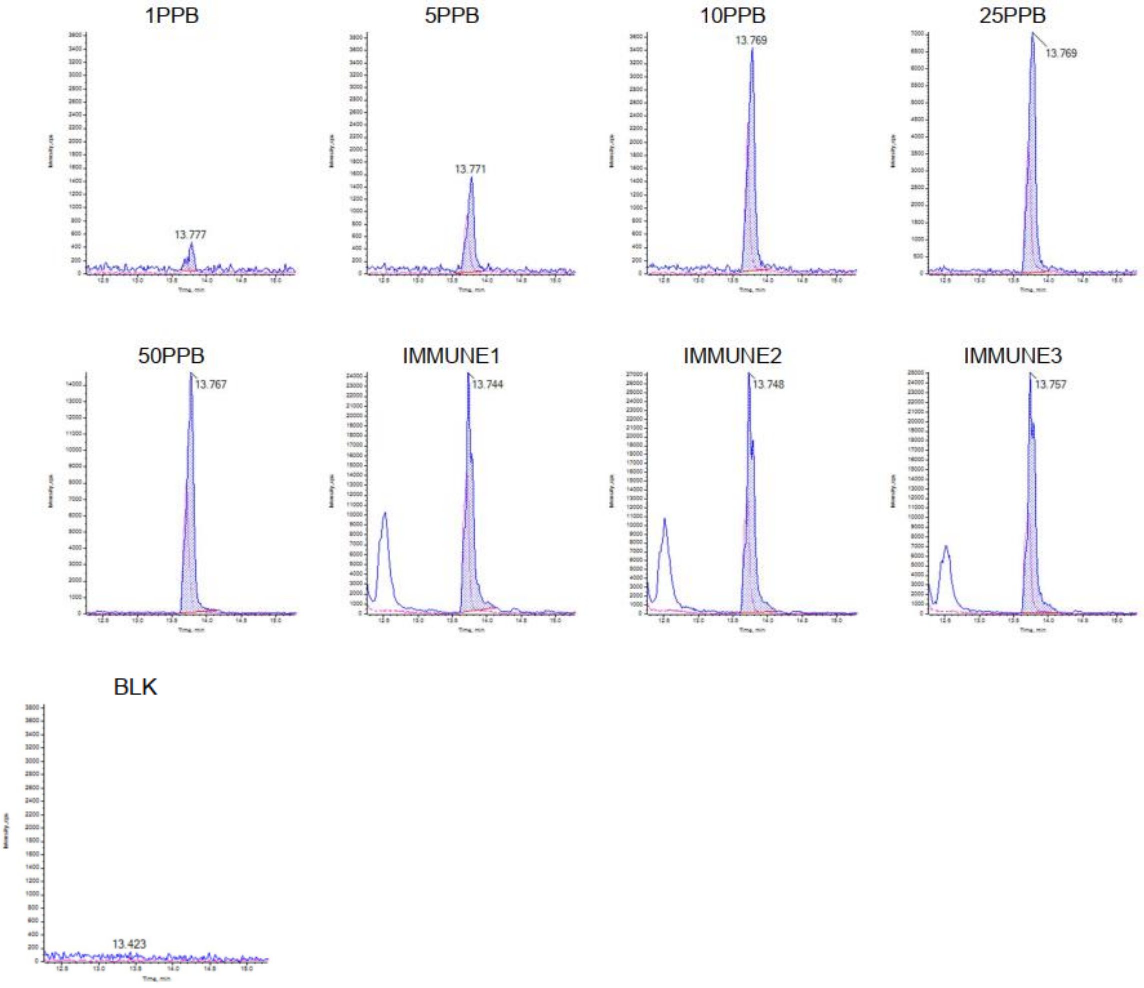
1PPB
13.777
5PPB
13.771
10PPB
13.769
25PPB
13.769
50PPB
13.767
IMMUNE1
13.744
IMMUNE2
13.748
IMMUNE3
13.757
BLK
13.423

(C)

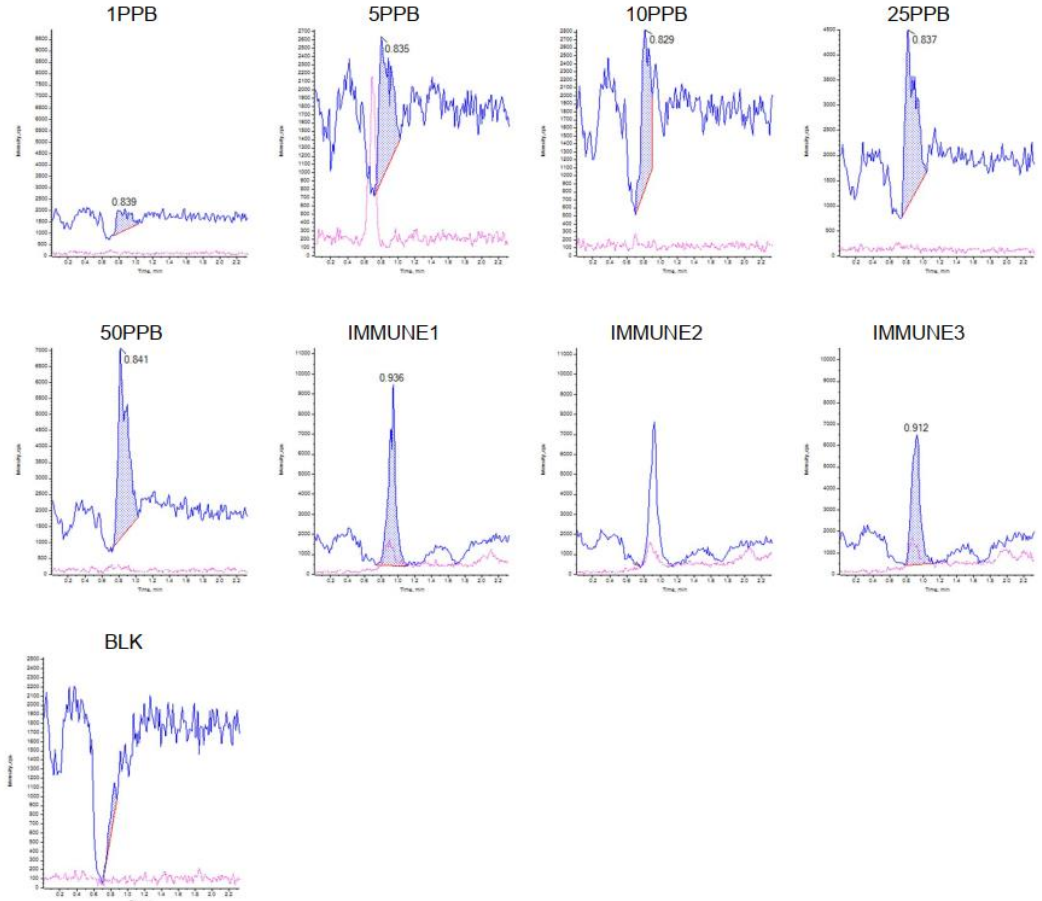
1PPB
0.839
5PPB
0.835
10PPB
0.829
25PPB
0.837
50PPB
0.841
IMMUNE1
0.936
IMMUNE2
IMMUNE3
0.912
BLK

Fig. S1

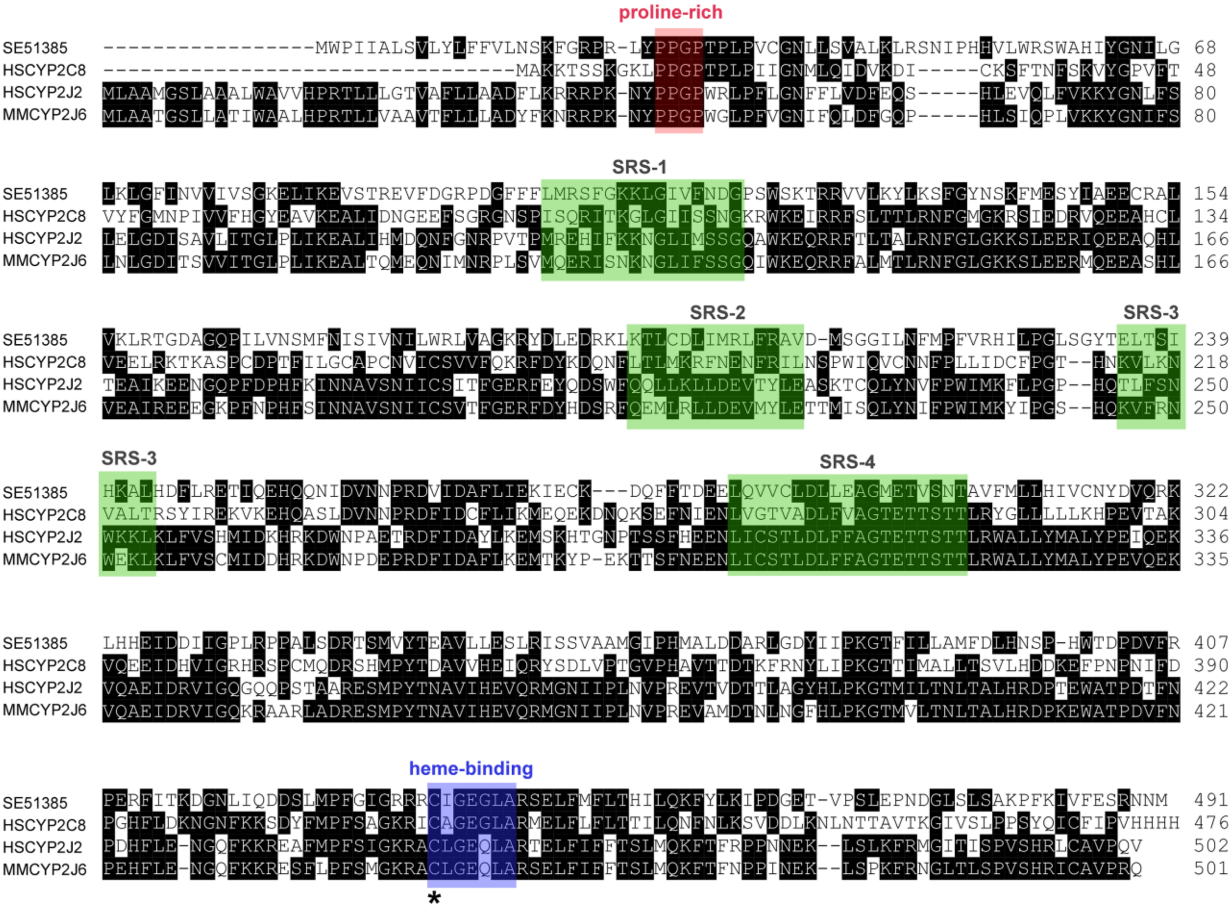
proline-rich
SE51385 -----------------MWPIIALSVLYLFFVLNSKFGRPR-LYPPGPTPLPVCGNLLSVALKLRSNIPHHVLWRSWAHIYGNILG 68
HSCYP2C8 ---------------------------------MAKKTSSKGKLPPGPTPLPIIGNMLQIDVKDI-----CKSFTNFSKVYGPVFT 48
HSCYP2J2 MLAAMGSLAAALWAVVHPRTLLLGTVAFLLAADFLKRRRPK-NYPPGPWRLPFLGNFFLVDFEQS-----HLEVQLFVKKYGNLFS 80
MMCYP2J6 MLAATGSLLATIWAALHPRTLLVAAVTFLLLADYFKNRRPK-NYPPGPWGLPFVGNIFQLDFGQP-----HLSIQPLVKKYGNIFS 80
SRS-1
SE51385 LKLGFINVVIVSGKELIKEVSTREVFDGRPDGFFFLMRSFGKKLGIVFNDGPSWSKTRRVVLKYLKSFGYNSKFMESYIAEECRAL 154
HSCYP2C8 VYFGMNPIVVFHGYEAVKEALIDNGEEFSGRGNSPISQRITKGLGIISSNGKRWKEIRRFSLTTLRNFGMGKRSIEDRVQEEAHCL 134
HSCYP2J2 LELGDISAVLITGLPLIKEALIHMDQNFGNRPVTPMREHIFKKNGLIMSSGQAWKEQRRFTLTALRNFGLGKKSLEERIQEEAQHL 166
MMCYP2J6 LNLGDITSVVITGLPLIKEALTQMEQNIMNRPLSVMQERISNKNGLIFSSGQIWKEQRRFALMTLRNFGLGKKSLEERMQEEASHL 166
SRS-2
SRS-3
SE51385 VKLRTGDAGQPILVNSMFNISIVNILWRLVAGKRYDLEDRKLKTLCDLIMRLFRAVD-MSGGILNFMPFVRHILPGLSGYTELTSI 239
HSCYP2C8 VEELRKTKASPCDPTFILGCAPCNVICSVVFQKRFDYKDQNFLTLMKRFNENFRILNSPWIQVCNNFPLLIDCFPGT--HNKVLKN 218
HSCYP2J2 TEAIKEENGQPFDPHFKINNAVSNIICSITFGERFEYQDSWFQQLLKLLDEVTYLEASKTCQLYNVFPWIMKFLPGP--HQTLFSN 250
MMCYP2J6 VEAIREEEGKPFNPHFSINNAVSNIICSVTFGERFDYHDSRFQEMLRLLDEVMYLETTMISQLYNIFPWIMKYIPGS--HQKVFRN 250
SRS-3
SRS-4
SE51385 HKALHDFLRETIQEHQQNIDVNNPRDVIDAFLIEKIECK---DQFFTDEELQVVCLDLLEAGMETVSNTAVFMLLHIVCNYDVQRK 322
HSCYP2C8 VALTRSYIREKVKEHQASLDVNNPRDFIDCFLIKMEQEKDNQKSEFNIENLVGTVADLFVAGTETTSTTLRYGLLLLLKHPEVTAK 304
HSCYP2J2 WKKLKLFVSHMIDKHRKDWNPAETRDFIDAYLKEMSKHTGNPTSSFHEENLICSTLDLFFAGTETTSTTLRWALLYMALYPEIQEK 336
MMCYP2J6 WEKLKLFVSCMIDDHRKDWNPDEPRDFIDAFLKEMTKYP-EKTTSFNEENLICSTLDLFFAGTETTSTTLRWALLYMALYPEVQEK 335
SE51385 LHHEIDDIIGPLRPPALSDRTSMVYTEAVLLESLRISSVAAMGIPHMALDDARLGDYIIPKGTFILLAMFDLHNSP-HWTDPDVFR 407
HSCYP2C8 VQEEIDHVIGRHRSPCMQDRSHMPYTDAVVHEIQRYSDLVPTGVPHAVTTDTKFRNYLIPKGTTIMALLTSVLHDDKEFPNPNIFD 390
HSCYP2J2 VQAEIDRVIGQGQQPSTAARESMPYTNAVIHEVQRMGNIIPLNVPREVTVDTTLAGYHLPKGTMILTNLTALHRDPTEWATPDTFN 422
MMCYP2J6 VQAEIDRVIGQKRAARLADRESMPYTNAVIHEVQRMGNIIPLNVPREVAMDTNLNGFHLPKGTMVLTNLTALHRDPKEWATPDVFN 421
heme-binding
SE51385 PERFITKDGNLIQDDSLMPFGIGRRRCIGEGLARSELFMFLTHILQKFYLKIPDGET-VPSLEPNDGLSLSAKPFKIVFESRNNM 491
HSCYP2C8 PGHFLDKNGNFKKSDYFMPFSAGKRICAGEGLARMELFLFLTTILQNFNLKSVDDLKNLNTTAVTKGIVSLPPSYQICFIPVHHHH 476
HSCYP2J2 PDHFLE-NGQFKKREAFMPFSIGKRACLGEQLARTELFIFFTSLMQKFTFRPPNNEK--LSLKFRMGITISPVSHRLCAVPQV 502
MMCYP2J6 PEHFLE-NGQFKKRESFLPFSMGKRACLGEQLARSELFIFFTSLMQKFTFNPPINEK--LSPKFRNGLTLSPVSHRICAVPRQ 501
*

Fig. S2

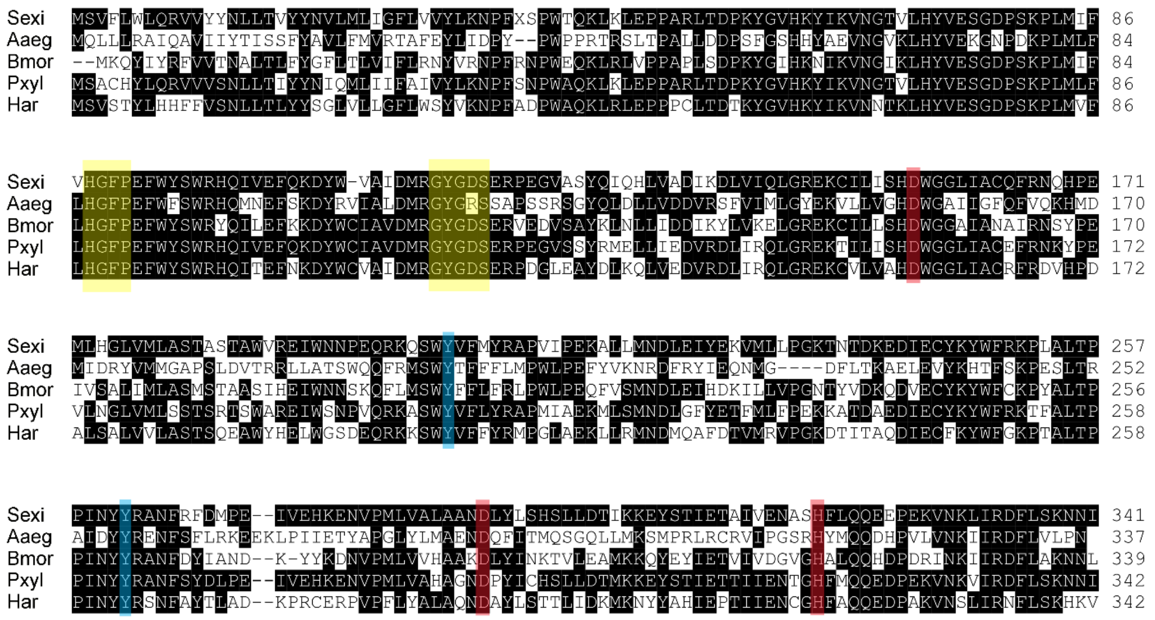
Sexi MSVFLWLQRVVYYNLLTVYYNVLMLIGFLVVYLKNPFXSPWTQKLKLEPPARLTDPKYGVHKYIKVNGTVLHYVESGDPSKPLMIF 86
Aaeg MQLLLRAIQAVIIYTISSFYAVLFMVRTAFEYLIDPY--PWPPRTRSLTPALLDDPSFGSHHYAEVNGVKLHYVEKGNPDKPLMLF 84
Bmor --MKQYIYRFVVTNALTLFYGFLTLVIFLRNYVRNPFRNPWEQKLRLVPPAPLSDPKYGIHKNIKVNGIKLHYVESGDPSKPLMIF 84
Pxyl MSACHYLQRVVVSNLLTIYYNIQMLIIFAIVYLKNPFSNPWAQKLKLEPPARLTDPKYGVHKYIKVNGTVLHYVESGDPSKPLMLF 86
Har MSVSTYLHHFFVSNLLTLYYSGLVLLGFLWSYVKNPFADPWAQKLRLEPPPCLTDTKYGVHKYIKVNNTKLHYVESGDPSKPLMVF 86
Sexi VHGFPEFWYSWRHQIVEFQKDYW-VAIDMRGYGDSERPEGVASYQIQHLVADIKDLVIQLGREKCILISHDWGGLIACQFRNQHPE 171
Aaeg LHGFPEFWFSWRHQMNEFSKDYRVIALDMRGYGRSSAPSSRSGYQLDLLVDDVRSFVIMLGYEKVLLVGHDWGAIIGFQFVQKHMD 170
Bmor LHGFPEFWYSWRYQILEFKKDYWCIAVDMRGYGDSERVEDVSAYKLNLLIDDIKYLVKELGREKCILLSHDWGGAIANAIRNSYPE 170
Pxyl LHGFPEFWYSWRHQIVEFQKDYWCIAVDMRGYGDSERPEGVSSYRMELLIEDVRDLIRQLGREKTILISHDWGGLIACEFRNKYPE 172
Har LHGFPEFWYSWRHQITEFNKDYWCVAIDMRGYGDSERPDGLEAYDLKQLVEDVRDLIRQLGREKCVLVAHDWGGLIACRFRDVHPD 172
Sexi MLHGLVMLASTASTAWVREIWNNPEQRKQSWYVFMYRAPVIPEKALLMNDLEIYEKVMLLPGKTNTDKEDIECYKYWFRKPLALTP 257
Aaeg MIDRYVMMGAPSLDVTRRLLATSWQQFRMSWYTFFFLMPWLPEFYVKNRDFRYIEQNMG----DFLTKAELEVYKHTFSKPESLTR 252
Bmor IVSALIMLASMSTAASIHEIWNNSKQFLMSWYFFLFRLPWLPEQFVSMNDLEIHDKILLVPGNTYVDKQDVECYKYWFCKPYALTP 256
Pxyl VLNGLVMLSSTSRTSWAREIWSNPVQRKASWYVFLYRAPMIAEKMLSMNDLGFYETFMLFPEKKATDAEDIECYKYWFRKTFALTP 258
Har ALSALVVLASTSQEAWYHELWGSDEQRKKSWYVFFYRMPGLAEKLLRMNDMQAFDTVMRVPGKDTITAQDIECFKYWFGKPTALTP 258
Sexi PINYYRANFRFDMPE--IVEHKENVPMLVALAANDLYLSHSLLDTIKKEYSTIETAIVENASHFLQQEEPEKVNKLIRDFLSKNNI 341
Aaeg AIDYYRENFSFLRKEEKLPIIETYAPGLYLMAENDQFITMQSGQLLMKSMPRLRCRVIPGSRHYMQQDHPVLVNKIIRDFLVLPN 337
Bmor PINYYRANFDYIAND--K-YYKDNVPMLVVHAAKDLYINKTVLEAMKKQYEYIETVIVDGVGHALQQHDPDRINKIIRDFLAKNNL 339
Pxyl PINYYRANFSYDLPE--IVEHKENVPMLVAHAGNDPYICHSLLDTMKKEYSTIETTIIENTGHFMQQEDPEKVNKVIRDFLSKNNI 342
Har PINYYRSNFAYTLAD--KPRCERPVPFLYALAQNDAYLSTTLIDKMKNYYAHIEPTIIENCGHFAQQEDPAKVNSLIRNFLSKHKV 342

Fig. S3

C1 (TPPU)

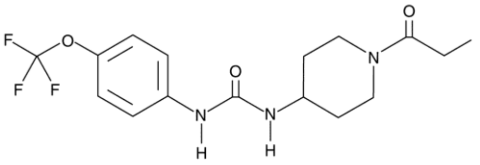

C2 (PTUPB)

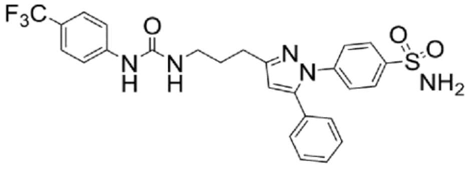

C3 (AUDA)

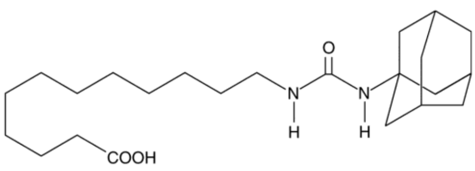

C4 (AEPU)

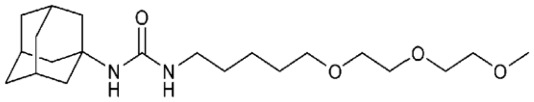

C5 (*t*-AUCB)

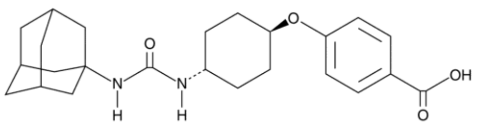

C6 (*t*-TUCB)

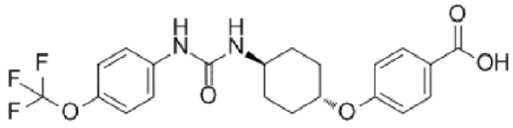

C7 (*c*-TUCB)

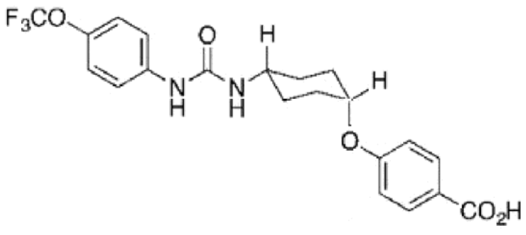

C8 (*c*-AUCB)

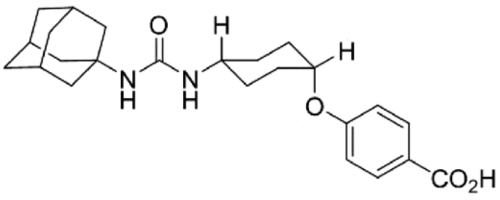

Fig. S4

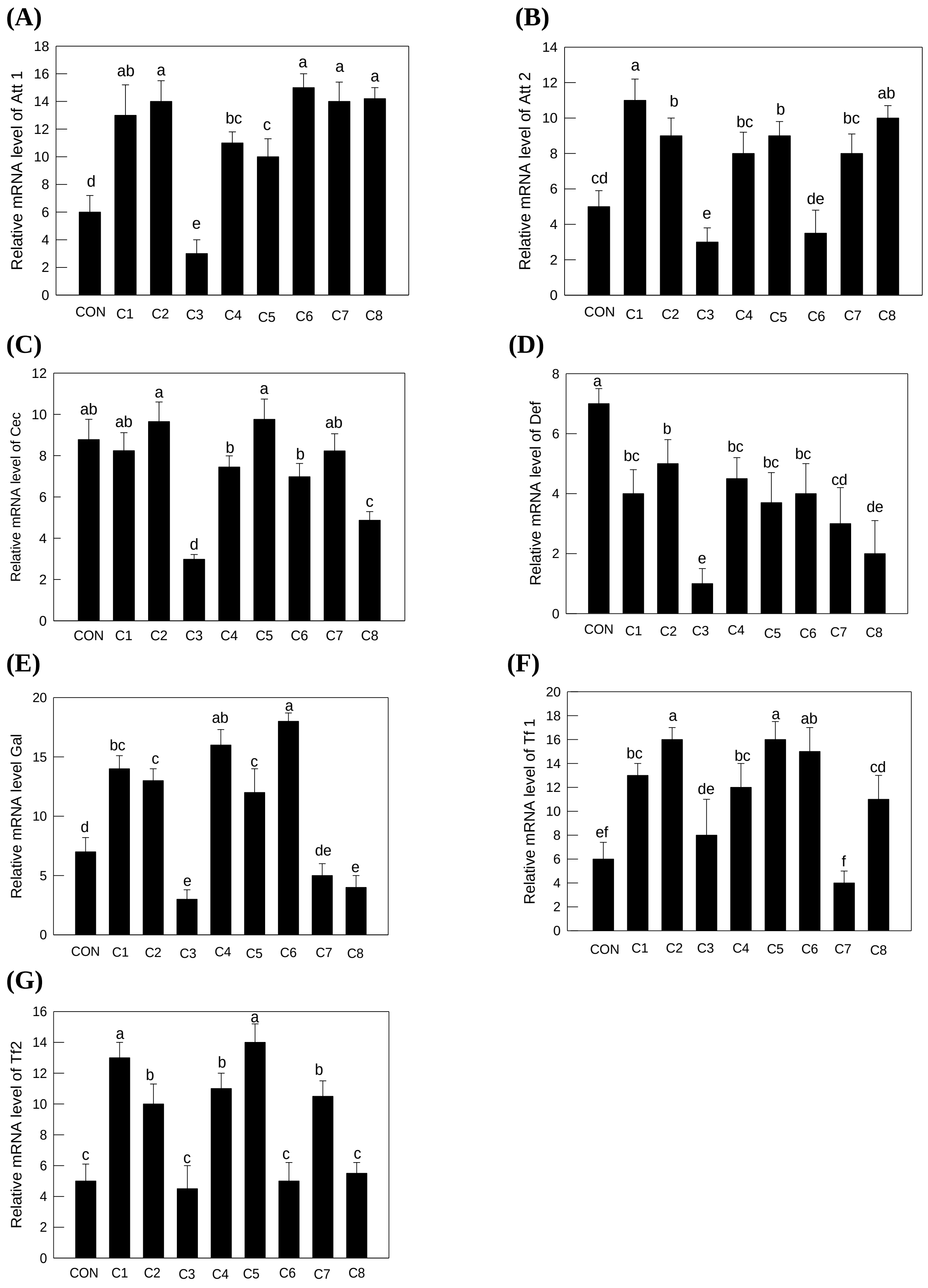
(A)
Relative mRNA level of Att 1
CON C1 C2 C3 C4 C5 C6 C7 C8
(B)
Relative mRNA level of Att 2
CON C1 C2 C3 C4 C5 C6 C7 C8
(C)
Relative mRNA level of Cec
CON C1 C2 C3 C4 C5 C6 C7 C8
(D)
Relative mRNA level of Def
CON C1 C2 C3 C4 C5 C6 C7 C8
(E)
Relative mRNA level Gal
CON C1 C2 C3 C4 C5 C6 C7 C8
(F)
Relative mRNA level of Tf 1
CON C1 C2 C3 C4 C5 C6 C7 C8
(G)
Relative mRNA level of Tf2
CON C1 C2 C3 C4 C5 C6 C7 C8

Fig. S5

(A) Figure 5A

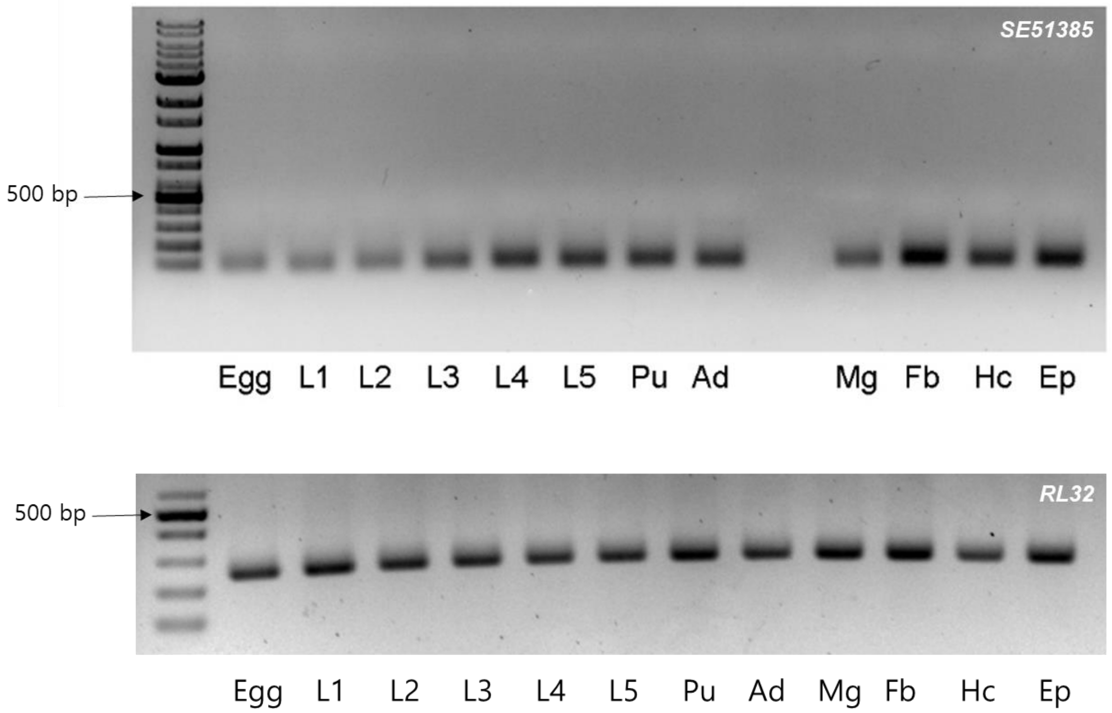

(B) Figure 8A

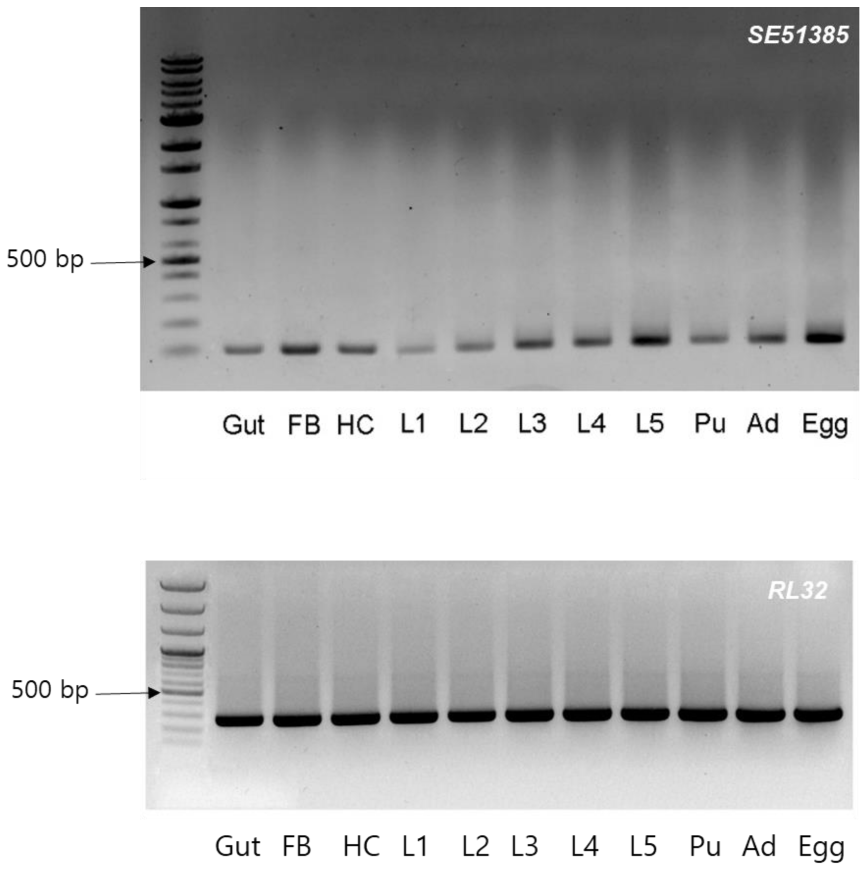

Fig. S6
